# Genetic Variants in the Bone Morphogenic Protein Gene Family Modify the Association between Residential Exposure to Traffic and Peripheral Arterial Disease

**DOI:** 10.1371/journal.pone.0152670

**Published:** 2016-04-15

**Authors:** Cavin K. Ward-Caviness, Lucas M. Neas, Colette Blach, Carol S. Haynes, Karen LaRocque-Abramson, Elizabeth Grass, Elaine Dowdy, Robert B. Devlin, David Diaz-Sanchez, Wayne E. Cascio, Marie Lynn Miranda, Simon G. Gregory, Svati H. Shah, William E. Kraus, Elizabeth R. Hauser

**Affiliations:** 1 Duke Molecular Physiology Institute, Duke University Medical Center, Durham, North Carolina 27710, United States of America; 2 Institute of Epidemiology II, Helmholtz Center Munich, Neuherberg, Germany 85764; 3 National Health and Environmental Effects Research Laboratory, US Environmental Protection Agency, Chapel Hill, North Carolina 27514, United States of America; 4 National Center for Geospatial Medicine, Rice University, Houston, Texas 77005, United States of America; 5 Division of Cardiovascular Medicine, Duke University Medical Center, Durham, North Carolina 27710, United States of America; 6 Department of Biostatistics and Bioinformatics, Duke University Medical Center, Durham, North Carolina 27710, United States of America; 7 Cooperative Studies Program Epidemiology Center-Durham, Veterans Affairs Medical Center, Durham, North Carolina 27701, United States of America; Vanderbilt University Medical Center, UNITED STATES

## Abstract

There is a growing literature indicating that genetic variants modify many of the associations between environmental exposures and clinical outcomes, potentially by increasing susceptibility to these exposures. However, genome-scale investigations of these interactions have been rarely performed particularly in the case of air pollution exposures. We performed race-stratified genome-wide gene-environment interaction association studies on European-American (EA, N = 1623) and African-American (AA, N = 554) cohorts to investigate the joint influence of common single nucleotide polymorphisms (SNPs) and residential exposure to traffic (“traffic exposure”)—a recognized vascular disease risk factor—on peripheral arterial disease (PAD). Traffic exposure was estimated via the distance from the primary residence to the nearest major roadway, defined as the nearest limited access highways or major arterial. The rs755249-traffic exposure interaction was associated with PAD at a genome-wide significant level (P = 2.29x10^-8^) in European-Americans. Rs755249 is located in the 3’ untranslated region of *BMP8A*, a member of the bone morphogenic protein (BMP) gene family. Further investigation revealed several variants in BMP genes associated with PAD via an interaction with traffic exposure in both the EA and AA cohorts; this included interactions with non-synonymous variants in *BMP2*, which is regulated by air pollution exposure. The BMP family of genes is linked to vascular growth and calcification and is a novel gene family for the study of PAD pathophysiology. Further investigation of *BMP8A* using the Genotype Tissue Expression Database revealed multiple variants with nominally significant (P < 0.05) interaction P-values in our EA cohort were significant BMP8A eQTLs in tissue types highlight relevant for PAD such as rs755249 (tibial nerve, eQTL P = 3.6x10^-6^) and rs1180341 (tibial artery, eQTL P = 5.3x10^-6^). Together these results reveal a novel gene, and possibly gene family, associated with PAD via an interaction with traffic air pollution exposure. These results also highlight the potential for interactions studies, particularly at the genome scale, to reveal novel biology linking environmental exposures to clinical outcomes.

## Introduction

Given the more than 255 million registered highway vehicles in the United States [1], http://www.bts.gov/publications/national_transportation_statistics/html/table_01_11.htmltraffic-related air pollution is a ubiquitous environmental exposure. Air pollution in general, and traffic-related air pollution in particular, is associated with adverse cardiovascular disease outcomes, including peripheral arterial disease (PAD) [2]. PAD is characterized by occlusive atherosclerosis in the peripheral arteries, principally the lower extremities, and affects approximately 4.6% of the population [3]. Complications from PAD include limb ischemia, infection, gangrene, and peripheral limb amputation, and PAD is a predictor of both all-cause and cardiovascular mortality [4]. Often estimated via the distance between a primary residence and the nearest higher-use roadway, residential exposure to traffic-related air pollution (”traffic exposure”) is associated with increased circulating angiogenic cells[5], PAD [6,7], deep vein thrombosis [8], incident coronary heart disease [9], and mortality [10,11].

PAD has a strong genetic component [12,13], and studies show that gene-environment interactions play a role in cardiovascular disease risk [14]. These gene-environment interactions can arise from a number of biological models. Ottman outlined five models that together encompass the possible biological underpinnings of gene-environment interactions, along with observed examples for each model [15]. All of these models could manifest as a traditional statistical multiplicative interaction and perhaps the most relevant model for air pollution is her “model B” where a genotype exacerbates the effect of a risk factor on a clinical outcome, e.g. genotypes exacerbating the effect of traffic air pollution on PAD. This interaction model has been previously shown to be the case with air pollution–GSTM1 variants and number of clinical outcomes [16,17]. However, despite a clear biological basis for gene-environment interactions and several observed gene-air pollution interactions few to no genome-wide interaction studies have been done.

To date the study of gene-environment interactions and PAD has been limited. In a 2008 study, a genetic variant in a gene cluster linked to smoking behavior was also linked to PAD and lung cancer [18]. However there have been no genome-scale efforts to estimate the joint effect of genetic variants and air pollution exposure on PAD, or even vascular disease in general. In this study, we examined the joint impact of traffic exposure and genetic variants on PAD risk at a genome-wide scale within the CATHeterization GENetics (CATHGEN) biorepository [19]. Our aim was to advance the understanding of PAD pathogenesis by using a genome-wide interaction study (GWIS) to analyze single nucleotide polymorphism (SNP)-traffic exposure interactions and thereby identify novel genes associated with PAD pathogenesis.

## Methods

### Study design

The CATHGEN cohort is a large sample and data biorepository of consenting patients receiving services at the Duke University Cardiac Catheterization Laboratory. A complete description is provided elsewhere [19]. Briefly, collection of the samples began in 2001 and was finished in 2011 with 9,334 unique patients enrolled over the 10 year period. In addition to the Health and Physical examination, demographic characteristics and peripheral blood was collected for subsequent analyses. The Duke University Institutional Review Board approved the collection and all subsequent analyses of the CATHGEN cohort. Clinical data was obtained from the Health and Physical examination performed by clinician prior to the catheterization procedure and supplemented by information from the medical record. The binary PAD variable indicated the presence or absence of PAD history, and it was collected during the Health and Physical examination. Clinical covariates separated by race for the CATHGEN cohort are presented in **Table 1**. Clinical covariates separated by PAD status are presented in **S1 Table**.

**Table 1:** Clinical covariates for the CATHGEN cohort (a), air pollution study cohort (b), and GWIS cohort (c).

Table 1				
1a. CATHGEN Clinical Covariates	All (N = 9334)	EA (6981)	AA (1778)	P
Age (SD)	60.8 (12)	62.1 (11.9)	56.9 (11.7)	< 0.001
BMI (SD)	30 (7.19)	29.5 (6.8)	31.8 (8.2)	< 0.001
Sex (% Female)	3531 (37.8)	2430 (34.8)	863 (48.5)	< 0.001
Smoking (% Ever Smoke)	4439 (47.6)	3407 (48.8)	751 (42.2)	< 0.001
Diabetes (% Yes)	2640 (28.3)	1767 (25.3)	702 (39.5)	< 0.001
Hypertension (% Yes)	6277 (67.2)	4507 (64.6)	1370 (77.1)	< 0.001
Dyslipidemia (% Yes)	5557 (59.5)	4305 (61.7)	931 (52.4)	< 0.001
PAD (% Yes)	709 (7.6)	552 (7.91)	119 (6.69)	0.005
1b. Air Pollution Study Cohort	All (N = 6066)	EA (4073)	AA (1363)	P
Age (SD)	61 (12)	62.2 (11.9)	56.9 (11.6)	< 0.001
BMI (SD)	30.1 (7.29)	29.6 (6.89)	32 (8.27)	< 0.001
Sex (% Female)	2333 (38.5)	1656 (35.2)	677 (49.7)	< 0.001
Smoking (% Ever Smoke)	2946 (48.6)	2355 (50.1)	591 (43.4)	< 0.001
Diabetes (% Yes)	1749 (28.8)	1204 (25.6)	545 (40)	< 0.001
Hypertension (% Yes)	4150 (68.4)	3074 (65.4)	1076 (78.9)	< 0.001
Dyslipidemia (% Yes)	3659 (60.3)	2922 (62.1)	737 (54.1)	< 0.001
PAD (% Yes)	478 (7.88)	380 (8.08)	98 (7.19)	0.31
1c. GWIS Cohort	All (N = 2177)	EA (1623)	AA (554)	P
Age (SD)	60 (12)	61.2 (11.9)	56.3 (11.4)	< 0.001
BMI (SD)	30.4 (7.34)	29.8 (7.02)	32.1 (7.99)	< 0.001
Sex (% Female)	936 (43)	639 (39.4)	297 (53.6)	< 0.001
Smoking (% Ever Smoke)	1051 (48.3)	815 (50.2)	236 (42.6)	< 0.001
Diabetes (% Yes)	665 (30.5)	428 (26.4)	237 (42.8)	< 0.001
Hypertension (% Yes)	1491 (68.5)	1045 (64.4)	446 (80.5)	< 0.001
Dyslipidemia (% Yes)	1286 (59.1)	987 (60.8)	299 (54)	0.005
PAD (% Yes)	138 (6.34)	104 (6.41)	34 (6.14)	0.90

**Table 1:** Relevant clinical covariates for the CATHGEN clinical cohort are summarized below for the entire cohort, the air pollution study cohort, and the GWIS cohort. These clinical covariates are also stratified by race, European-Americans (EA) and African-Americans (AA). P-values were assessed via ANOVA for the continuous covariates of Age and BMI and were assessed via a Chi-squared test for the binary covariates Sex, Smoking, Diabetes, Hypertension, Dyslipidemia, and PAD.

### Residential Exposure to Traffic Assessment

**Fig 1:**
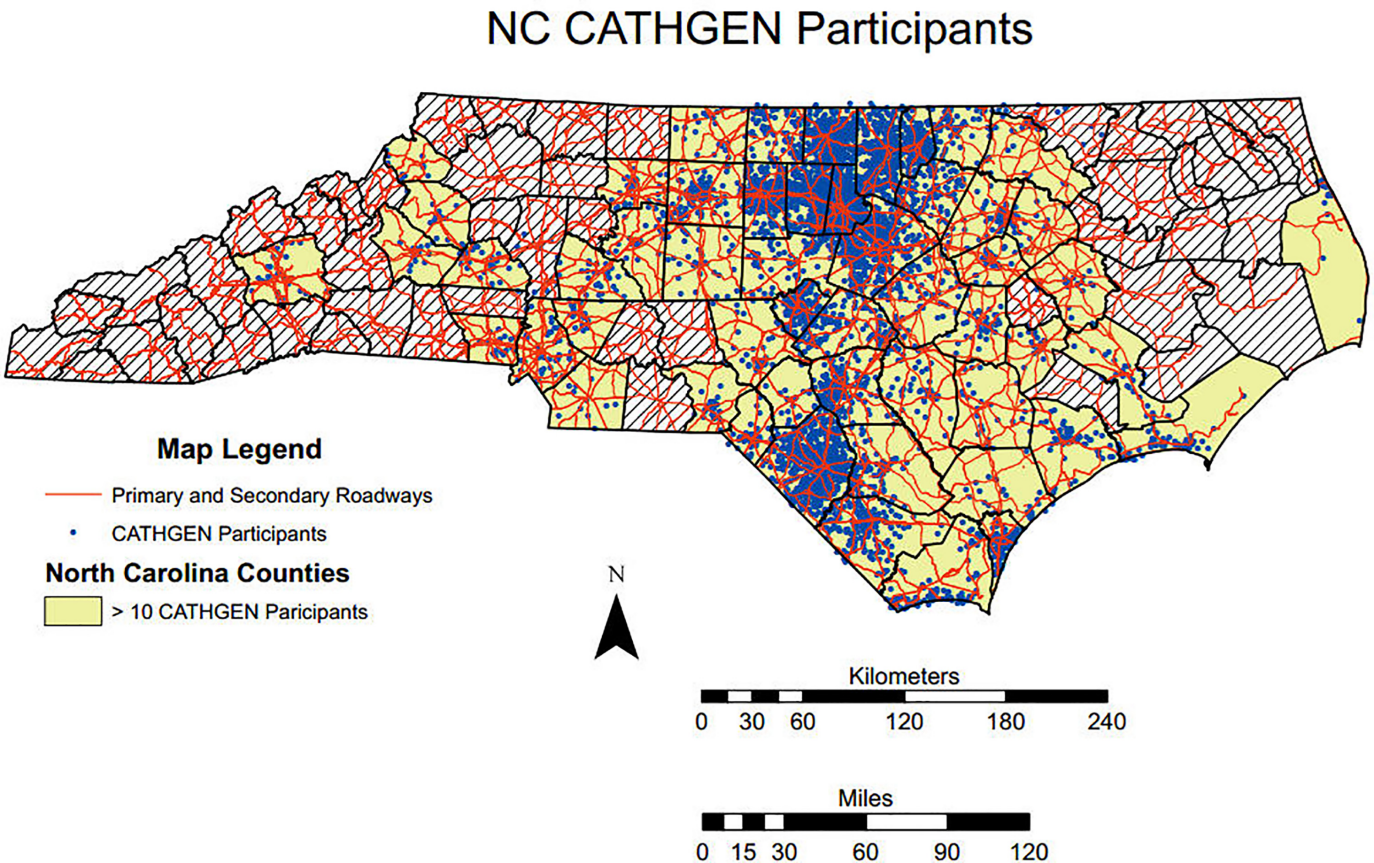
CATHGEN participant Locations. The location of the North Carolina CATHGEN participants who were selected for these analyses. The locations have been randomized to a small degree to protect the identity of the individuals while maintaining the spatial structure and dependency in the data.

The National Center for Geospatial Medicine, previously at Duke University and now at Rice University, performed assignment of residential geocodes using the patient addresses. Geocoded primary residential address information was obtained for a total of 8,071 CATHGEN participants, 7,158 residing in North Carolina. We restricted all analyses to CATHGEN participants residing in North Carolina to enhance the homogeneity of the sample, for example excluding individuals who may have traveled from long distances for specialized treatment at Duke University. This restriction matches previous approaches taken with the CATHGEN cohort [20]. After geocoding the participants, we used the ArcGIS [21] software package to import both the patient locations and locations of all primary and secondary roadways in North Carolina (**Fig 1**) and to calculate the perpendicular distance between each primary residence and the nearest primary (A1) or secondary (A2) roadway, as defined by the North Carolina Department of Transportation [22]. Primary roadways were defined as limited-access highways with interchanges while secondary roadways were inter- and intra-city arterials that had multiple lanes and potentially at-grade intersections. This definition is consistent with the definition used for the Master Address File/Toplogically Integrated Geographic Encoding and Referencing Feature Class Code employed by the U.S. Census Bureau [23]. This distance to nearest roadway measure of traffic exposure strongly correlates with exposure to particulate matter generated by traffic [24] and is associated with health outcomes [7,8,25]. Full details of the geocoding, restriction to those individuals residing in North Carolina, and calculation of traffic exposure via the distance between the primary residence and nearest major roadway have been previously described [26].

### Genotyping

Genotyping was performed on 3,512 CATHGEN participants using the Illumina HumanOmni 1-Quad_v1-0_C array. The selection of patients for genotyping was done irrespective of their geocoded location and yielded a total of 2,177 individuals (1623 European-Americans (EA), 554 African-Americans (AA) residing in North Carolina and possessing genome-wide genotype data (**Table 1C**). Quality control was performed prior to all analyses and matched previous quality control for race-stratified genome-wide association studies performed in CATHGEN [27]. The quality control included removal of related individuals, low quality genotypes, SNPs with a call frequency < 98%, individuals with a call rate < 98%, and individuals whose genotypic gender did not match the recorded self-reported gender. Genome-wide interaction study analyses were restricted to those SNPs with a minor allele frequency (MAF) greater than 0.05. At total of 905,956 variants passed QC in at least one of the two race-stratified cohorts and were thus available for analysis.

### Statistical methods

**Fig 2:**
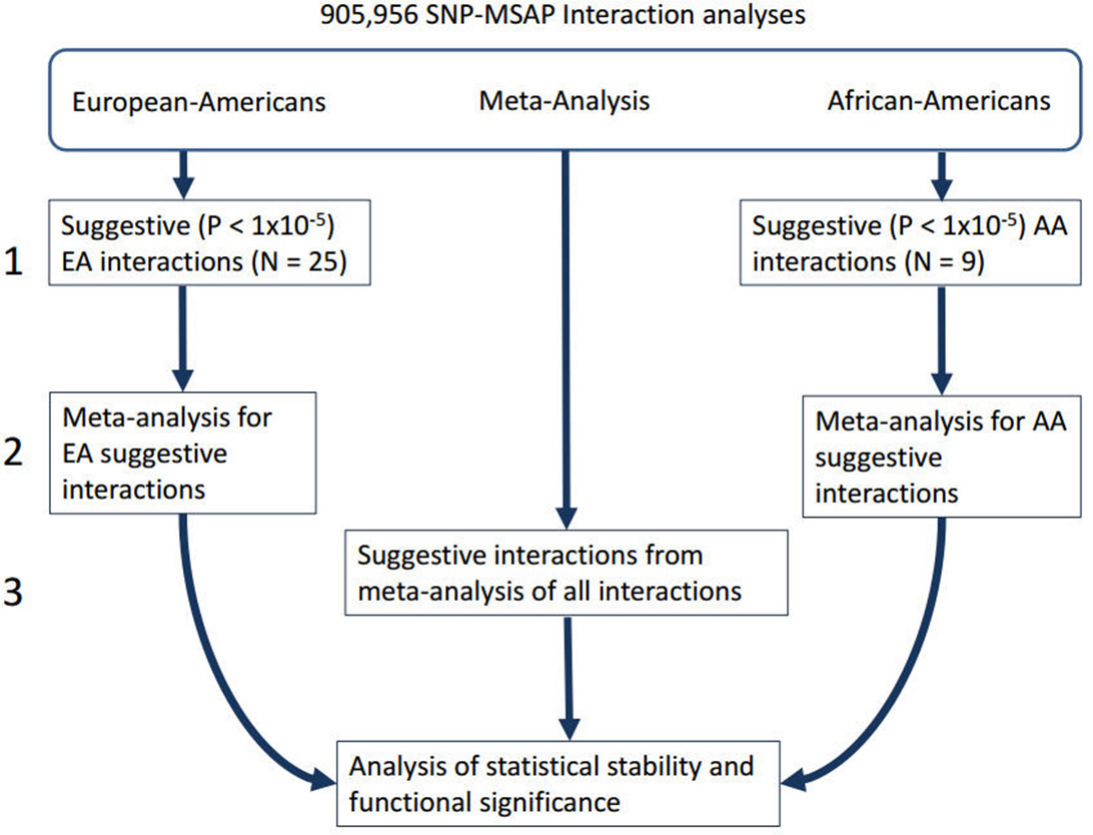
Flowchart displaying the analysis plan undertaken. In the first step separate SNP-traffic exposure GWIS of the EA and AA cohorts was performed. Following this suggestive (P < 1x10^-5^) results were examined for replication via a meta-analysis of both cohorts using METAL[31]. Finally step 3 involved an examination of suggestive results from a meta-analysis of all the interactions.

All statistical analyses were conducted using the R statistical package [28]. The statistical analysis consisted of three stages (**Fig 2**). The first stage was a race stratified analysis of the European-American (EA) and African-American (AA) cohorts. Case-control logistic regression was used to calculate the odds ratio for the SNP-traffic exposure interaction term and a Score test [29] was used to calculate the significance of this odds ratio. An additive genetic model was used for all analyses, with a multiplicative interaction for the SNP-traffic exposure term. For the traffic exposure measure the distance between the primary residence and nearest roadway was scaled to the inter-quartile range as done for previous analyses [26], and for both the AA and EA cohorts the model was adjusted for age, sex, and principal components calculated using Eigenstrat [30] to remove racial substructure. Based on previous genome-wide association studies done with EA and AA within CATHGEN [27] we used principal components for the EA cohort and two principal components for the AA cohort. A clinical covariate adjusted-model that added body mass index, and binary indicators for hypertension, smoking, diabetes, and dyslipidemia was also used. However, the addition of these covariates did not substantially affect the interaction odds ratio as compared to the previous model and thus results from the more parsimonious age, sex, and racial principal components model were considered the primary results. Results from the clinical covariate-adjusted model for the interactions with P < 1x10^-4^ are presented in **S2 Table**.

A robust Score test was used to calculate the significance of the gene-environment interaction term. The quantile-quantile plot revealed inflation in the AA GWIS; thus, further adjustment of these results was performed using the genomic control F-test (GCF) whereby we generated new p-values based on the chi-squared statistic from the Score test. GCF is the method recommended for large-scale analyses where a small p-value is required [32]. We used 103,196 randomly selected SNPs to calculate the median of the Score test statistic, which provided a better adjustment than the mean of the Score statistic. The median of the Score statistics then was used to calculate the inflation in the AA GWIS (λ_m_ = 1.22). The Score test statistic was then adjusted by λ_m_ and an F-test (F(1,100)) was used to calculate the new P-value for each interaction, which is presented in the tables. To account for multiple testing of 905,956 interactions (one for each SNP) we used the conventional genome-wide significance of P < 5x10^-8^. We defined suggestive interactions as those with a P < 1x10^-5^ matching the threshold used in previous GWIS [33]; a nominal P-value of P < 0.05 was used for replication of results between the race-stratified GWIS and examinations of candidate genes uncovered by each GWIS.

The last two stages of the analysis used METAL [31] to conduct a fixed effect inverse-variance weighted meta-analysis of the most significant race-stratified GWIS results and a meta-analysis of all available interactions. For the meta-analyses the MAF cutoff was relaxed so that variants with a MAF ≥ 0.05 in at least one of the two cohorts would still be analyzed. Thus the second stage of the analysis was an examination of the suggestive race-stratified GWIS results after meta-analysis. The third stage of analysis was an examination of the results from the meta-analysis of all interactions to identify consistent results, in terms of the same direction of effect, across the race-stratified GWIS. After performing these three stages, the results were checked for their statistical stability and biological significance. We considered statistical stability by examining plots of the fitted residuals and estimated probabilities for each logistic regression analysis. Potential outliers were identified, removed, and the analyses rerun. An order of magnitude or greater change in the odds ratio or p-value after removal of outliers was considered evidence of statistical instability and statistically unstable results were not considered further. The biological significance of each SNP involved in the interaction was investigated by examining their annotation to known genes and the potential regulatory function of the sequence surrounding each SNP, e.g. alteration of CpG sites important for regulation via methylation or location in open chromatin regions. Information from the NCBI dbSNP database [34] and information on DNaseI hypersensitivity sites from the ENCODE project [35] were used to annotate variants with their biological significance Data on DNaseI hypersensitivity sites from the Duke University contributions to the ENCODE project are summarized at http://dnase.genome.duke.edu/ [36]. We used the Genotype Tissue Expression database Release V6 (GTEx) in order to determine if any variants found were known expression quantitative trait loci (eQTL) [37–39]. From the GTEx resource we report all single tissue cis-eQTLs with a q-value < 0.05 [37,40].

## Results

**Fig 3:**
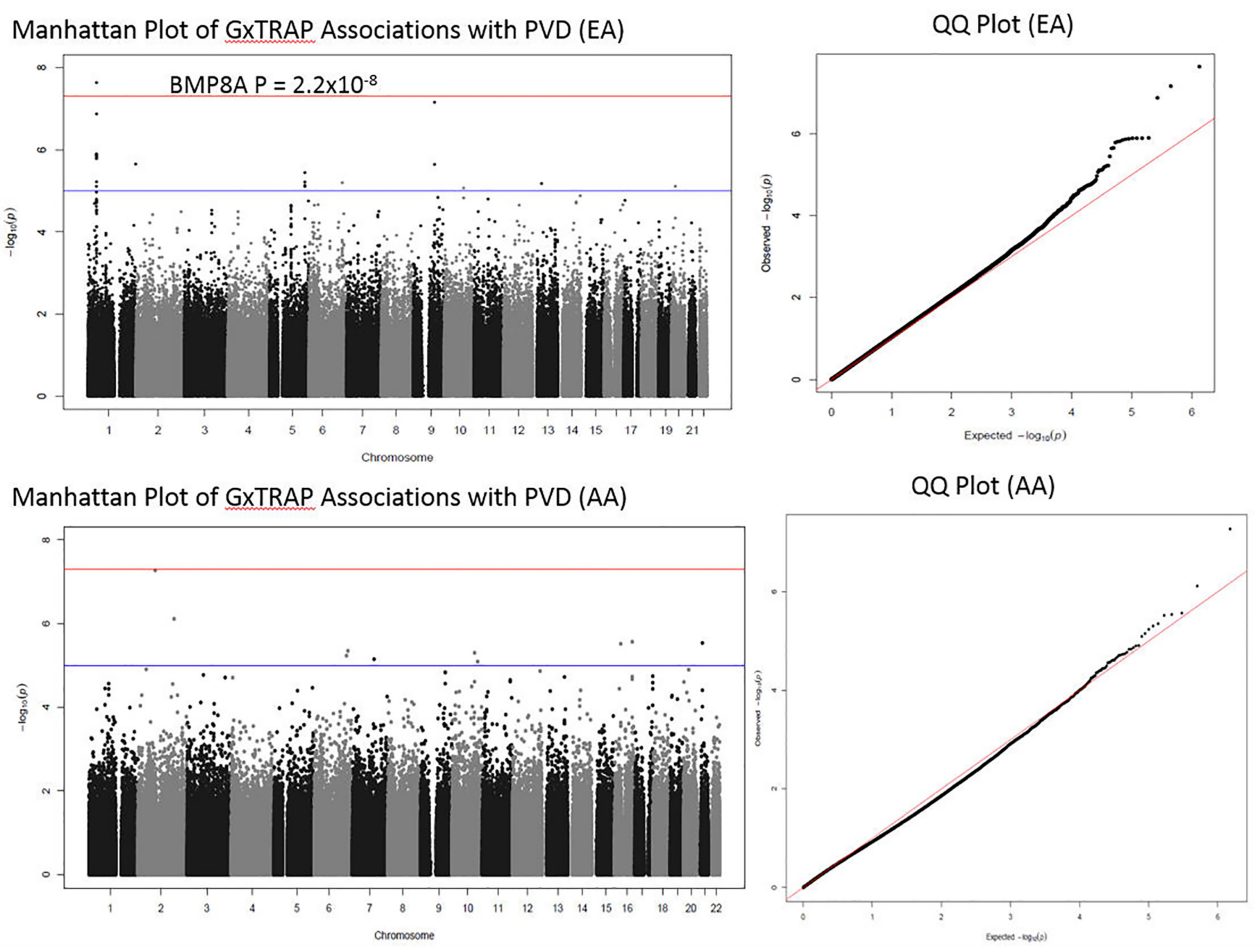
Manhattan and QQ plots for both the European-American (top) and African-American (bottom) cohorts. The red line in the Manhattan plots represents the Bonferroni significance level for 1,000,000 tests (P < 5x10^-8^) while the blue line represents a suggestive P (P < 1x10^-5^).

**Fig 4:**
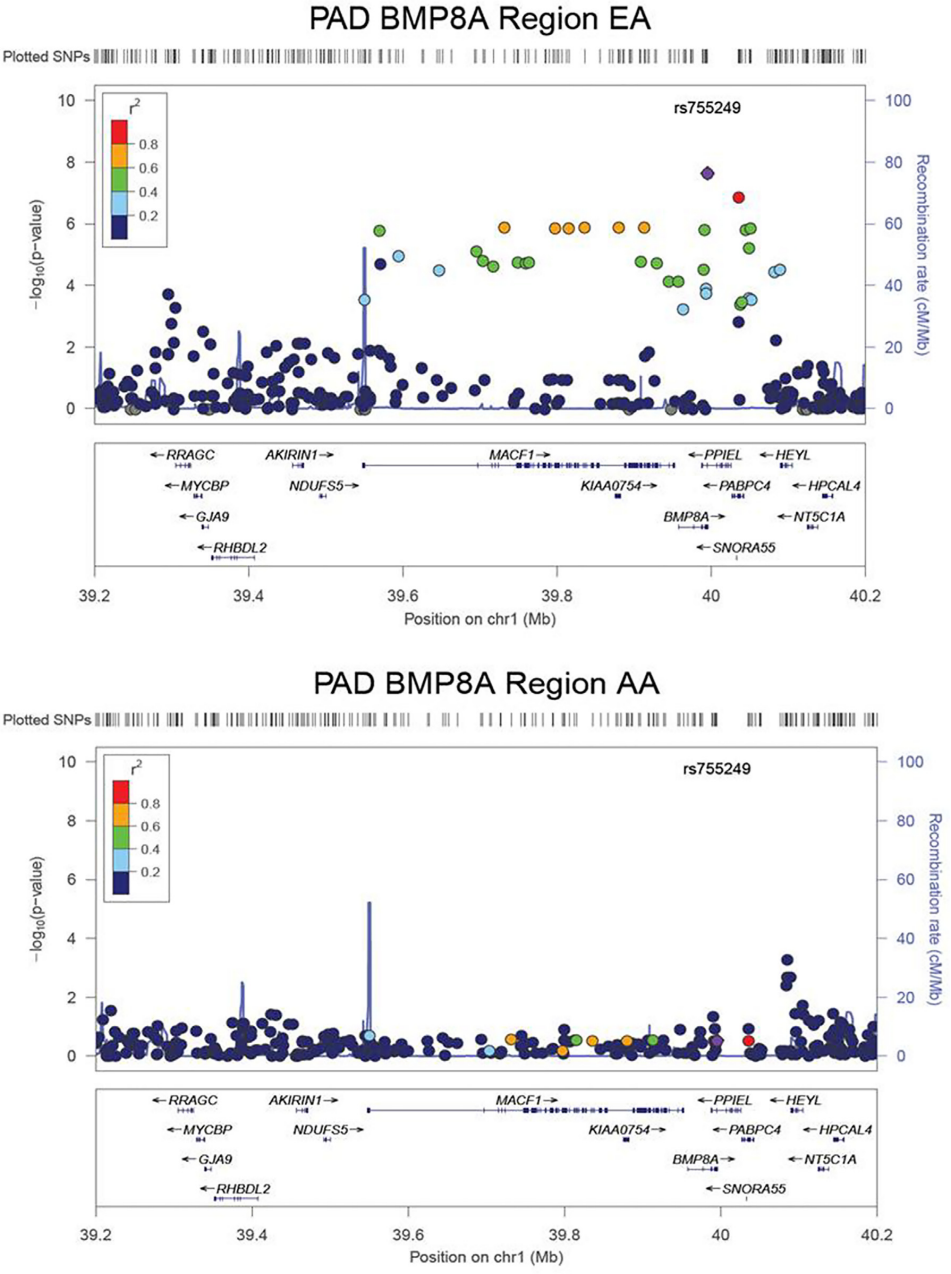
LocusZoom [41] plots of the BMP8A region. LocusZoom reveals strong LD between rs755249 in BMP8A and multiple SNPs in MACF1 among the European-Americans (top). This region has a much lower level of LD in the African-American cohort (bottom).

For the 6,066 individuals residing in North Carolina for whom clinical data available were also available, we calculated their traffic exposure according to the procedure defined in the Methods section (**Table 1B**). In these individuals an interquartile range decrease (IQR) in the distance to major roadways (641 meters) was significantly associated with a decrease in PAD prevalence in a race and sex adjusted logistic regression model (OR = 0.88, CI: 0.77–1.00, P = 0.044), which is consistent with results from previous studies [6,8]. For subjects on whom we also performed genome-wide genotyping via the Illumina HumanOmni 1-Quad v1-0 C array system, we investigated SNP-by-traffic exposure interactions associated with PAD in EA (N = 1623) and AA (N = 554) cohorts (**Table 1C**). The QQ-plot for both the EA and corrected AA GWIS did not reveal significant genomic inflation (**Fig 3**). For the EA GWIS, rs755249, located in the 3’-untranslated region (UTR) of *BMP8A*, achieved genome-wide significance (P = 2.3x10^-8^, **Table 2A**). As the AA MAF did not meet the > 5% cutoff (AA MAF = 0.04, EA MAF = 0.24), the rs755249-traffic exposure interaction was not considered stable in the race-stratified AA GWIS. In addition to the single genome-wide significant interaction there were 24 additional suggestive interactions in the EA GWIS (**S3 Table**), of which the top 10 ranked by p-value are presented in **Table 2A**. Fourteen of the 25 variants with interaction P < 1x10^-5^ were within 1Mb of *BMP8A*; these included six intronic and two missense SNPs in *MACF1*. The strong linkage disequilibrium (LD) across *BMP8A* and *MACF1* in EA individuals limited our ability to identify independent signals within the region using only the EA cohort (**Fig 4**). The *BMP8A* region had the typical “candlestick” pattern often observed for significant variants in genome-wide association studies. All interactions with P < 1x10^-4^ in the primary model are given in **S3 Table**. To place the interactions in the context of the genetic main effect and environmental effect the odds ratio, standard errors, and P-values for all three terms (interaction, genetic main effect, and traffic exposure) from the primary model are given in **S4 Table** for both the EA and AA GWIS.

**Table 2:** Suggestive (P < 1x10^-5^) interactions for the EA (a) and AA (b) GWIS.

**2a. EA Interactions with P < 1x10**^**-5**^ **(top 10 of 25 ordered by p-value)**											
Chrom	BP	SNP		OR (EA)	P (EA)	MAF (EA)	OR (AA)		P (AA)	MAF (AA)	Locus
1	39995074	rs755249 *		3.45	2.29E-08	0.24	0.04			< 0.05	*BMP8A*
9	97261572	rs9409787		4.93	6.87E-08	0.13	0.46		0.15	0.26	DHS
1	40035686	rs17513135		3.34	1.33E-07	0.23	0.05			< 0.05	*PAPBC4*
1	39731550	rs4660214		3.08	1.26E-06	0.22	0.12			< 0.05	*MACF1*
1	39913351	rs2296173		3.08	1.28E-06	0.22	0.12			< 0.05	*MACF1*
1	39835817	rs2296172		3.08	1.28E-06	0.22	0.12			< 0.05	*MACF1*
1	39880319	rs3768302		3.08	1.28E-06	0.22	0.12			< 0.05	*MACF1*
1	39815143	rs16826093		3.07	1.33E-06	0.22	0.12			< 0.05	*MACF1*
1	39797055	rs16826069		2.99	1.35E-06	0.22	0.66			< 0.05	*MACF1*
1	40050568	rs7539279		2.81	1.41E-06	0.34	0.94		0.85	0.49	
**2b. AA Interactions with P < 1x10**^**-5**^											
Chrom	BP		SNP	OR (AA)	P (AA)	MAF (AA)	OR (EA)	P (EA)		MAF (EA)	Locus
2	161732534		rs634138	6.1	7.67E-07	0.08	0.44			< 0.05	
16	78805200		rs2161719	5.76	2.72E-06	0.18	0.86	0.60		0.14	*WWOX*
21	21812972		rs2989314	9.79	2.90E-06	0.12	2.21	0.08		0.07	
16	27270607		rs9940555	7.72	3.03E-06	0.17	1.58			< 0.05	*NSMCE1*
6	151236180		rs9397365	21.07	4.46E-06	0.09	0.37	0.0054		0.15	*MTHFD1L*
10	101121718		rs11190074	7.36	4.97E-06	0.09	0.69	0.37		0.09	*CNNM1*
6	144749359		rs6570628	21.77	5.82E-06	0.12	1.62	0.32		0.05	*UTRN*
7	97231097		rs7787478	7.92	7.06E-06	0.13	0.47	0.09		0.10	
10	115070140		rs499832	6.66	8.09E-06	0.20	0.70	0.36		0.09	*HABP2*

**Table 2:** Associations with P < 1x10^-5^ for the EA and AA GWIS. SNPs were restricted to a minor allele frequency (MAF) of > 0.05. Variants with a MAF < 0.05 in one of the ethnicities are listed with “< 0.05” in the MAF column. Blank cells for the odds ratio, standard error, and P indicate that the model did not converge or the MAF was less than 0.05. Four interactions were removed from Table 2b due to being statistically unstable. DHS = DNaseI Hypersensitivity sites. Rs9409787 was in a site designated as a DNaseI hypersensitivity site in retinal endothelial cells. For the EA cohort only the top 10 results (25 total) are shown. The complete list of 25 variants involved in suggestive EA interactions appears in **S5 Table**.

* = genome-wide significant

None of the AA results achieved genome-wide significance after correcting for genomic inflation and statistical instability (**Table 2B**). The most significant, stable SNP-traffic exposure interaction was with rs634138 (P = 7.67x10^-7^) located in an intergenic region of chromosome 2. The nine suggestive AA interactions are presented in **Table 2B** after removing four interactions that were statistically unstable. Of the remaining nine suggestive interactions, six were with variants located in introns, and three were with intergenic variants. Examinations of the intergenic SNPs, rs634138 (P = 7.67x10^-7^), rs2989314 (P = 2.90x10^-6^), and rs7787478 (P = 7.06x10^-6^), revealed that only rs2989314 was located near genes (pseudo-genes *KRT18P2* and *RPS3AP1*). Examination of DNAseI hypersensitivity sites from multiple tissues via http://dnase.genome.duke.edu/ [35] did not indicate that any of the three variants were in a putative regulatory region as defined by DNAseI hypersensitivity sites. Of the suggestive EA interactions six had a consistent direction of association in the AA GWIS, but none of them replicated (P<0.05) in the AA GWIS (**Table 2, S5 Table**). Of the nine suggestive AA GWIS interactions two were consistent in the EA with the intergenic variant rs2989314 just nearly missing the P-value cutoff for replication (EA GWIS P = 0.08).

In stage two of the analysis, to more formally examine the consistency of the suggestive variants from the race-stratified GWIS analyses, we performed a meta-analysis of the EA and AA GWIS interactions with P < 1x10^-5^. As many variants differed in allele frequency between the EA and AA cohorts, we removed the MAF cutoff for this analysis. **Table 3A** gives the ten most significant interactions from the meta-analysis of the suggestive race-stratified GWIS results, with the full list given in **S6 Table**.

**Table 3:** Meta-Analysis results for suggestive (P<1x10^-5^) race-stratified interactions (a) and all interactions (b).

**Table 3a. Meta-analysis of suggestive EA and AA interactions (top 10 of 34)**												
**Chrom**	**BP**	**SNP**	**OR (EA)**	**P (EA)**	**MAF (EA)**	**OR (AA)**	**P (AA)**	**MAF (AA)**	**Meta-Analysis P**	**Consistent**	**Effect Allele**	**Locus**
5	162019898	rs6879255	2.68	6.06E-06	0.29	1.69	0.26	0.27	7.76E-06	Yes	G	
1	39995074	rs755249	3.45	2.29E-08	0.24	0.04	0.34	0.04	1.45E-05	No	G	*BMP8A*
5	161997490	rs10063408	2.83	3.57E-06	0.28	1.23	0.70	0.16	2.73E-05	Yes	G	
1	40044713	rs7520271	2.80	1.55E-06	0.34	0.99	0.98	0.50	3.22E-05	Yes	C	
1	40035686	rs17513135	3.34	1.33E-07	0.23	0.05	0.35	0.04	4.54E-05	No	G	*PABPC4*
1	40050568	rs7539279	2.81	1.41E-06	0.34	0.94	0.87	0.49	4.60E-05	No	G	
5	162023980	rs2431268	2.67	7.76E-06	0.29	1.18	0.68	0.34	4.79E-05	Yes	G	
1	39695155	rs10788933	2.76	7.72E-06	0.30	0.90	0.79	0.34	6.46E-05	Yes	G	*MACF1*
9	97261572	rs9409787	4.93	6.87E-08	0.13	0.46	0.19	0.26	6.76E-05	No	G	*NUTM2F*
13	40297797	rs9548897	2.76	6.61E-06	0.49	1.08	0.85	0.35	6.86E-05	Yes	G	*COG6*
**Table 3b. Suggestive interactions from meta-analysis of all variants**												
**Chrom**	**BP**	**SNP**	**OR (EA)**	**P (EA)**	**MAF (EA)**	**OR (AA)**	**P (AA)**	**MAF (AA)**	**Meta-Analysis P**	**Consistent**	**Effect Allele**	**Locus**
5	82260021	rs256811	10.80	0.002	0.03	3.87	8.02E-05	0.11	2.54E-06	Yes	G	
5	99157979	rs7448169	2.26	6.42E-05	0.45	2.80	0.02	0.28	3.78E-06	Yes	G	
1	183743599	rs12024301	3.63	0.003	0.05	5.05	1.01E-04	0.07	4.84E-06	Yes	C	*RGL1*
1	39989926	rs710913	2.42	3.04E-05	0.36	2.11	0.07	0.31	6.70E-06	Yes	G	*BMP8A*
5	162019898	rs6879255	2.68	6.06E-06	0.29	1.69	0.26	0.27	7.76E-06	Yes	G	

**Table 3:** Meta-analysis results from the suggestive EA and AA interactions (a) and all interactions (b). For the meta-analysis variants with a MAF < 0.05 in one of the two cohorts were allowed. For Table 3a only the 10 most significant results are presented with the full results appearing in **S6 Table**. The column consistent gives whether the effect was consistent after aligning the results so that both cohorts had the same effect allele (Effect Allele). The race-stratified odds ratios (OR (EA) and OR (AA)) are given relative to the minor allele for each race.

In the meta-analysis of all interactions, no meta-analysis result reached genome-wide significance; however there were five suggestive interactions. Examination of the suggestive meta-analysis results independent of their association in either race-stratified GWIS revealed an additional *BMP8A* SNP (rs710913, meta-analysis P = 6.70x10^-6^, 3’-UTR *BMP8A*, **Table 3B**). Of the remaining four meta-analysis associations three were intronic and rs12024301 was in an intron of *RGL1* (**Table 3B**).

Given the genome-wide significant interaction in *BMP8A* as well as the combined evidence for an additional *BMP8A* variant in the meta-analysis, we investigated further interaction in this gene. In the EA cohort, a closer examination of interactions with variants in *BMP8A* revealed five interactions in addition to the rs755249-traffic exposure interaction: the intronic SNPs rs710913 (P = 3.04x10^-5^); rs2004330 (P = 5.74x10^-4^); and the 3’-UTR variants rs1180341 (P = 1.24x10^-4^); rs1180343 (P = 1.74x10^-4^) and rs3738676 (P = 1.51x10^-6^). The rs710913-traffic exposure interaction was the most consistent association interaction of those in the BMP genes examined (P = 0.07 AA, P = 6.70x10^-6^ meta-analysis). This variant was the only variant in the *BMP8A* region with MAF > 0.05 in both cohorts (EA MAF = 0.36, AA MAF = 0.31) (**Table 3B**). We examined the LD patterns in the two racial groups. As shown in **S1 Fig**, substantial LD was observed between the typed *BMP8A* SNPs and rs755249 in the EA cohort (r^2^ between 0.23 and 0.55) with an r^2^ = 0.53 with rs710913 in the EA cohort. The LD was much weaker in the AA cohort (highest pairwise r^2^ = 0.08) which effectively separated the conjoined EA GWIS associations in this region and localized the consistent signal to *BMP8A*. Given our observed associations and previous evidence of an association between traffic-generated air pollution and *BMP2* expression [42], we examined traffic exposure interactions with SNPs in six genes in the BMP family: *BMP1*, *BMP2*, *BMP4*, *BMP8A*, *BMP9*, and *BMPER*. Among the EA interactions, there were five SNPs in *BMP2* with an interaction P < 0.05: a missense variant, rs235768 (R → S, P = 7.28x10^-3^); a synonymous variant, rs1049007 (S -> S, P = 0.011); and three intronic SNPs, rs235764 (P = 0.020), rs235767 (P = 0.017), and rs7270163 (P = 0.034). Of these five SNPs two had P < 0.05 in the AA cohort and consistent direction of association under meta-analysis, rs7270163 (AA P = 0.0025, meta-analysis P = 7.98x10^-4^) and rs235764 (AA P = 5.46x10^-3^, meta-analysis P = 6.36x10^-4^). None of the remaining BMP family genes examined had any SNPs with P < 0.05 and consistent direction of association in both cohorts. **S7 Table** lists the results for all analyzed interactions in BMP family genes.

In addition to examining genes belonging to the BMP family, we examined additional interactions in *MACF1* and *PABPC4*, two genes near *BMP8A* with strong interaction signals however no additional interaction in these genes achieved a P < 0.05 in either race-stratified GWIS.

To connect the interactions found in BMP family genes to gene expression we searched for these variants in Release V6 of the Genotype Tissue Expression database (GTEx)[37,38]. Given the association evidence we focused our eQTL investigation on variants with interaction P < 0.05 in *BMP8A* and *BMP2*. No variant in BMP2 showed evidence (q < 0.05) of being an eQTL in any of the 45 tissues with greater than 60 samples analyzed in GTEx. Variants in BMP8A with traffic exposure interaction P < 0.05 in the EA GWIS showed significant evidence (q < 0.05) of being an eQTL for *BMP8A* across several tissues (**Table 4**). Rs755249, the most significant variant, showed evidence for being a cis-eQTL for four different genes (**S8 Table**). *BMP8A* is purported to play a role in spermatogenesis [43,44] and all *BMP8A* variants were eQTLs in testis tissue (**Table 4**). However, rs3738676 and rs755249 were most significantly cis-eQTLs in tibial artery tissue for *RP11-69E11*.*4* (P = 6.5x10^-16^) and *OXCT2P1* (P = 2.4x10^-15^) respectively (**S8 Table**). When specifically examining *BMP8A* gene expression for each of the *BMP8A* variants we often found non-reproductive tissues among the significant eQTL tissues including tissue potentially relevant in PAD such as: tibial nerve (rs3738676, P = 1.2x10^-5^; rs2004330, P = 5.5x10^-7^; rs755249, P = 3.6x10^-6^, rs1180341, P = 2.6x10^-6^; rs1180343, P = 1.3x10^-6^), tibial artery (rs1180341, P = 5.3x10^-6^), subcutaneous adipose (rs3738676, P = 3.7x10^-6^; rs710913, P = 1.7x10^-5^), and atrial appendage (rs2004330, P = 1.3x10^-9^).

**Table 4:** eQTL associations for *BMP8A* variants.

*BMP8A* eQTLs		rs3738676	rs2004330	rs1180343	rs1180341	rs755249	rs710913
	Adipose—Subcutaneous	3.7x10^-6^ (0.37)					1.7x10^-5^ (-0.33)
	Artery—Tibial				5.3x10^-6^ (-0.36)		
	Brain—Cortex				3.7x10^-6^ (-0.44)		
	Esophagus–Muscularis		8.6x10^-6^ (-0.37)	1x10^-5^ (-0.38)	2.3x10^-6^ (-0.4)	1.5x10^-5^ (0.44)	5.5x10^-6^ (-0.38)
	Heart—Atrial Appendage		1.3x10^-9^ (-0.52)	1.2x10^-9^ (-0.54)	8.8x10^-10^ (-0.54)		
	Heart—Left Ventricle				6.6x10^-6^ (-0.42)		
	Nerve—Tibial	1.2x10^-5^ (0.20)	5.5x10^-7^ (-0.25)	1.3x10^-6^ (-0.24)	2.6x10^-6^ (-0.23)	3.6x10^-6^ (0.25)	
	Testis	2.1x10^-12^ (0.54)	6.9x10^-11^ (-0.50)	2.2x10^-10^ (-0.48)	2.3x10^-16^ (-0.61)	2x10^-7^ (0.5)	9.5x10^-12^ (-0.52)

**Table 4:** Significant (q < 0.05) single tissue *BMP8A* eQTL associations (via GTEx [37]) for those BMP8A variants with an interaction P < 0.05 in the EA cohort. Only cis (±1Mb) eQTLs are included in GTEx database. The p-value for the eQTL association is given with the effect size according to GTEx in parentheses.

## Discussion

In this first genome-wide gene-environment interaction study for PAD we have uncovered several suggestive interactions and one genome-wide significant interaction that indicates that a spectrum of genetic variants modify the association between PAD and residential exposure to traffic. The genome-wide significant interaction was found in the EA GWIS with a variant located in *BMP8A* and this region was highly represented among the suggestive interactions. Figures demonstrating the interactions for SNPs rs755249 and rs710913 are included in **S2 Fig** and **S3 Fig**. Using the results from the AA GWIS we were able to narrow down the large LD block in the EA, which spanned the nearby genes of *MACF1* and *PABPC4* (**S1 Fig**) and localize our interaction signal to *BMP8A* as it was the only gene with a consistent interaction with P < 0.05 in both cohorts.

Genes in the BMP family are regulators of muscle mass [45], are involved in endothelial signaling pathways[46,47], and affect vascular smooth muscle cell progression [48]. They promote vascular, aortic, and smooth muscle cell calcification[49–52], and are associated with atherosclerosis [50,53] and angiogenesis [54,55]. To date *BMP8A* has been primarily implicated in spermatogenesis and development of the epididymis [43,44]. Methylation is proposed to be an important regulator of *BMP8A* [56,57]. Genetic variants in *BMP8A* were significant eQTLs in a variety of tissues perhaps most prominently tibial nerve where five variants in *BMP8A* were associated with *BMP8A* expression (**Table 4**). Two of the six *BMP8A* variants examined in the GTEx database, rs3738676 and rs755249, were most prominently eQTLs in tibial artery tissue, a tissue highly relevant for PAD. The other four variants examined were additionally often strongly associated as a cis-eQTL in non-reproductive tissue types (**S8 Table**). Taken together we conclude that while *BMP8A* expression is significantly regulated by non-coding variants in reproductive tissue, *BMP8A* expression is also significantly regulated in a variety of other tissues that point to novel functions of this gene. Additionally, variants annotated to *BMP8A* may broadly regulate cis-genes in a variety of tissue types. Further research on this genetic locus is needed to fully elucidate the role of *BMP8A* and the potentially regulatory variants.

In our analysis, the five most significant *BMP8A* SNPs in the EA cohort altered a CpG site (**Table 5**) with rs755249 interrupting a CpG dimer (CGC**G** -> CGC**A)** and rs710913 removing a CpG site (**C**GGA -> **T**GGA). Given the evidence that traffic-related air pollution alters DNA methylation status [58–60], the important role that epigenetics and methylation play in the vascular endothelium[61], and the link between DNA methylation and vascular diseases [62–64] it is reasonable to speculate that the causal pathway linking traffic exposure, *BMP8A*, and PAD runs through DNA methylation events.

**Table 5:** Sequence surrounding the five most significant *BMP8A* variants in the EA GWIS.

SNP	P (EA)	P (AA)	Sequence
rs755249	2.29E-08	0.34	CGC**G** → CGC**A**
rs3738676	1.51E-06	0.35	CTC**G**→CTC**T**
rs710913	3.04E-05	0.05	CC**C**G→CC**T**G
rs1180341	1.24E-04	0.64	AT**C**G→AT**T**G
rs1180343	1.74E-04	0.43	TC**C**G→TC**T**G

**Table 5:** The variant is given in bold in the “Sequence” column.

In previous studies, *BMP2* gene expression increased in vascular endothelial cells after exposure to black carbon, a by-product of incomplete combustion in internal combustion engines [42]. *BMP2* is associated with calcification in vascular cells [48] and may mediate the effects of estrogen-related receptor γ on vascular calcification[52]. Thus, our study adds to the growing body of evidence linking *BMP2*, air pollution, and vascular dysregulation. The other BMP genes have a variety of functions related to bone and vascular growth; however none of the other BMP family genes showed a level of association near that of *BMP2* or *BMP8A*, and only *BMP2* is linked to air pollutants specifically associated with both traffic-related air pollution and vascular phenotypes. *BMP2* also was the only BMP gene to have coding variants associated with PAD via an interaction with traffic exposure; both a synonymous (rs1049007, P = 0.01) and a missense (rs235768, R → S, P = 7.27x10^-3^) variant showed nominal association in the EA cohort. While neither of these variants was an eQTL for *BMP2* in GTEx, we believe that further research is warranted given the possible dependence on air pollution exposure.

In addition *BMP8A* variants, we also observed interactions with SNPs in the nearby genes of *MACF1* and *PABPC4*. These two genes in combination with *BMP8A* bridge an extended locus with significant LD in the EA cohort (**Fig 4**). An examination of the LocusZoom [41] plots reveals that the p-values in the EA cohort correlate strongly with the LD with our most significant variant, rs755249. This would fit a single locus hypothesis where associations in this genetic region are due to LD with a single causal locus that is associated with PAD via an interaction with traffic exposure, and rs755249 is a marker variant for that locus. This hypothesis is supported by the complete lack of interactions in *MACF1* or *PABPC4* with even a nominal level of significance, i.e. P < 0.05, in the AA GWIS where the LD was much lower, while we observed an additional interaction with P < 0.05 in the AA GWIS in *BMP8A* (**Table 3B**).

For the AA GWIS there were relatively few suggestive interactions, indicative of the lower power due to the smaller sample size. Among the suggestive interactions was rs2161716, located in an intron of *WWOX*. A previous genome-wide interaction study of gene-smoking interactions associated with coronary artery calcification replicated an interaction with a variant in *WWOX* [33]. It was hypothesized that this could be due to the involvement of *WWOX* and other replicated interactions in inflammatory processes mediated by the NF-κB pathway. As the NF-κB pathway mediates bone remodeling [65], this adds to our evidence that variants more typically associated with bone regulation may be involved in vascular disease pathogenesis via interactions with environmental exposures. Of the remaining suggestive AA interactions none have been previously associated with vascular disease. *UTRN* is a target for microRNA-206 which has been associated with skeletal muscle development [66] and microRNAs are known to be associated with air pollution exposure [67]. *HABP2* is a Hyaluronan binding protein associated with acute lung injury [68], and inflammation from lung injury is hypothesized to be a causal mechanism linking air pollution exposures and vascular disease [2]. Additionally *HABP2* is sometimes called Factor VII-activating protein and is involved in the activation of Factor VII and fibrinolysis [68]. Several hemostatic factors have been associated with PAD [69] implying a potential role for *HABP2* in PAD pathogenesis potentially mediated or modified by air pollution exposure as indicated by our results.

There are some limitations of this study related to sample size, exposure bias, statistical instability, and generalizability. The primary limitation is sample size. Relative to estimated sample sizes for interaction studies, both of these GWIS are small; this likely limited our ability to find more than one genome-wide significant variant. To combat this, we used a Score test rather than the traditional Wald test to determine associations. The Score test is the most powerful asymptotic test under many conditions, and thus increased our power to detect significant results [70].

Although distance to roadways is a well-utilized and recognized measure in the air pollution epidemiology literature [25,26,71] and is associated with PAD in our cohort and others [6], it remains a composite measure of all components of traffic-related pollution: particulate, gaseous, and even noise pollution. Thus, in this study, it is impossible to identify the specific causal components of traffic exposure. Nevertheless, we believe that this limitation is mitigated by the robust nature of this measure. Additionally, distance is measured in the same manner and with the same error for all individuals no matter their residential location minimizing potential bias due to differential exposure assessment methods and measurement accuracies. We were able to maximize our sample size relative to other methods that can suffer a loss of sample size due to several factors, such as air pollution monitors not being active or measurements being incompatible due to differing measurement methods. Future studies might incorporate specific components of traffic generated air pollution to determine the causal traffic exposure components.

The statistical stability of results is a concern for any study, particularly large unbiased studies such as this one. We addressed this issue by evaluating significant associations for statistical stability and removing unstable results. If our observed p-value or odds ratio changed by an order or magnitude or more after the removal of an apparent outlier that result was not considered stable. We also removed results with standard errors greater than 10, as standard error of that magnitude combined with a small p-value meant the estimated odds ratio was beyond the realm of what might be considered reasonable.

At 6.3% (**Table 1**) the prevalence of PAD in our GWIS cohort is higher than the estimated 4.6% prevalence for the general population [3]. This slight enrichment of PAD cases is likely secondary to sampling bias from a group presenting for assessment of coronary vascular disease, to which PAD is related. This unique sampling nature of CATHGEN can limit generalizability. To address this, we evaluated the consistency of the associations across ethnicities and have highlighted several trans-ethnicity consistent interactions. Associations consistent across ethnicities may be more generalizable than ethnicity specific associations. Nevertheless, it is important that our observations be replicated in other general population-based studies.

## Conclusion

We observed that a decrease in the proximity from a primary residence to major roadways is associated with an increased prevalence of PAD, and this association is modified by genetic variants. The rs755249-traffic exposure interaction was associated with PAD at a genome-wide significant level in the EA cohort. Rs755249 had a very low minor allele frequency in the AA cohort (MAF = 0.04); thus, this interaction could not be replicated in the AA GWIS. In addition to rs755249, we observed multiple additional associations in *BMP8A*, including rs710913—the most significant SNP in the BMP gene family in our meta-analysis. Both rs755249 and rs710913 are potentially eQTLs for *BMP8A* and other nearby genes in tissues related to the vasculature and peripheral limbs (**Table 4**). An examination of other genes in the BMP gene family revealed important *BMP2* interactions with traffic exposure. The potential for novel functions for *BMP8A* and for multiple genes in the BMP gene family to be associated with PAD via interactions with traffic exposure highlight the need for more experimental models and cohort analyses to confirm and expand upon these findings.

## Supporting Information

S1 Fig **Plot of the linkage disequilibrium in both the EA (a) and AA (b) cohorts.** We see different patterns of LD between the EA and AA cohorts for BMP8A. The European-Americans show a higher degree of LD than the African-Americans. Cells are colored according to the r^2^ with darker cells indicating a higher r^2^.
(PDF)

S2 Fig Interaction plot for rs75529 for EA (left) and AA (right) GWIS. This figure shows the interaction between rs755249 and traffic exposure as associated with PAD in the EA and AA. On the x-axis is the number of minor alleles and on the y-axis is the predicted probability of PAD as given by our primary model. For the age term the average age in EA (61.2 y) and AA (56.3 y) was used and the sex was assumed to be male. For the principal components the average for each of the race-specific principal components was used. The colors correspond to the distance from primary residence to the nearest major roadway given in meters.
(PDF)

S3 Fig Interaction plot for rs710913 for EA (left) and AA (right) GWIS. This figure shows the interaction between rs710913 and traffic exposure as associated with PAD in the EA and AA. On the x-axis is the number of minor alleles and on the y-axis is the predicted probability of PAD as given by our primary model. For the age term the average age in EA (61.2 y) and AA (56.3 y) was used and the sex was set to be male for both plots. For the principal components the race-specific average for each principal component was used. The colors correspond to the distance from primary residence to the nearest major roadway given in meters. We see a consistent effect for the interaction for both the EA and AA GWIS with the effect somewhat attenuated in the AA cohort which is consistent with the reduced p-value for the rs710913-traffic exposure interaction in this cohort.
(PDF)

S1 Table Clinical Covariates and PVD Status. Clinical covariates for the CATHGEN cohort for the full cohort (a), the air pollution study cohort (b), and the genome-wide interaction study (GWIS) cohort (c). The GWIS cohort represents those CATHGEN participants used for this analysis.
(PDF)

S2 Table Clinical model GWIS Results. Results from the clinical factor adjusted model for variants with a P < 1x10^-4^ in this model. The model was adjusted for age, sex, BMI, hypertension, diabetes, smoking status, and hyperlipidemia. The model also included terms for the genetic main effect, environmental main effect, and SNP-traffic exposure interaction term. Results shown are those for the interaction term.
(PDF)

S3 Table Primary model GWIS Results. Results from the primary GWIS model which adjusted for age, sex, and race-specific principal components to remove population substructure for all interactions with a P < 1x10^-4^. In addition to age and sex, the model also included terms for the genetic main effect, environmental main effect, and SNP-traffic exposure interaction term. Results shown are those for the interaction term.
(PDF)

S4 Table **Effect Estimates for interaction, main effect, and environmental association terms for suggestive EA (a) and AA (b) GWIS variants.** The odds ratio (OR), standard error (SE), and P-value (P) for the SNP-traffic exposure interaction (GxE), genetic (SNP) main effect (G), and traffic exposure (E) terms for the primary model for both the EA (a) and AA (b) suggestive (P < 1x10^-5^) variants.
(PDF)

S5 Table Suggestive interactions from the EA GWIS. The full list of all 25 suggestive interaction (P < 1x10^-5^) interactions from the EA GWIS.
(PDF)

S6 Table Meta-analysis of the suggestive interactions from the race-stratified GWIS. The full list of the meta-analysis results from all 34 suggestive interactions (P < 1x10^-5^) interactions from the race-stratified GWIS.
(PDF)

S7 Table Interaction results for all loci tested in the bone morphogenic protein family of genes (BMP1, BMP2, BMP4, BMP7, BMP8A, BMP9, BMP10, and BMPER). (PDF)

S8 Table Cis-eQTL associations for all genes associated with variants in BMP8A (EA GWIS P < 0.05). This table gives the eQTL associations for all genes associated with variants in BMP8A with an interaction P < 0.05 in the EA GWIS. Data on the eQTLs was taken from the GTEx Release V6[37].
(PDF)
