# Supplementary figures and images for: Genetic Variants in the Bone Morphogenic Protein Gene Family Modify the Association between Residential Exposure to Traffic and Peripheral Arterial Disease
Source: PLoS One. 2016 Apr 15;11(4):e0152670. doi: 10.1371/journal.pone.0152670 (PMC4833382; doi:10.1371/journal.pone.0152670)

(a) EA BMP8A LD

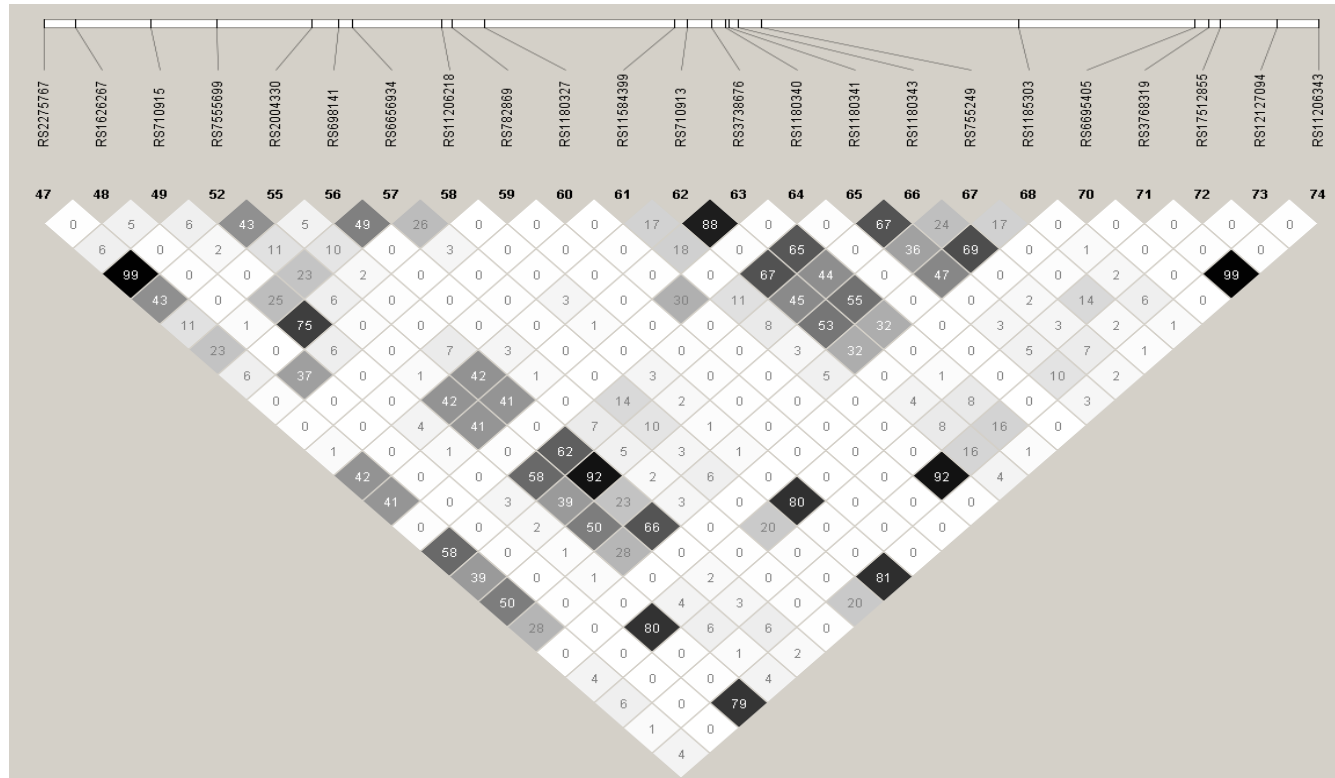

(b) AA BMP8A LD

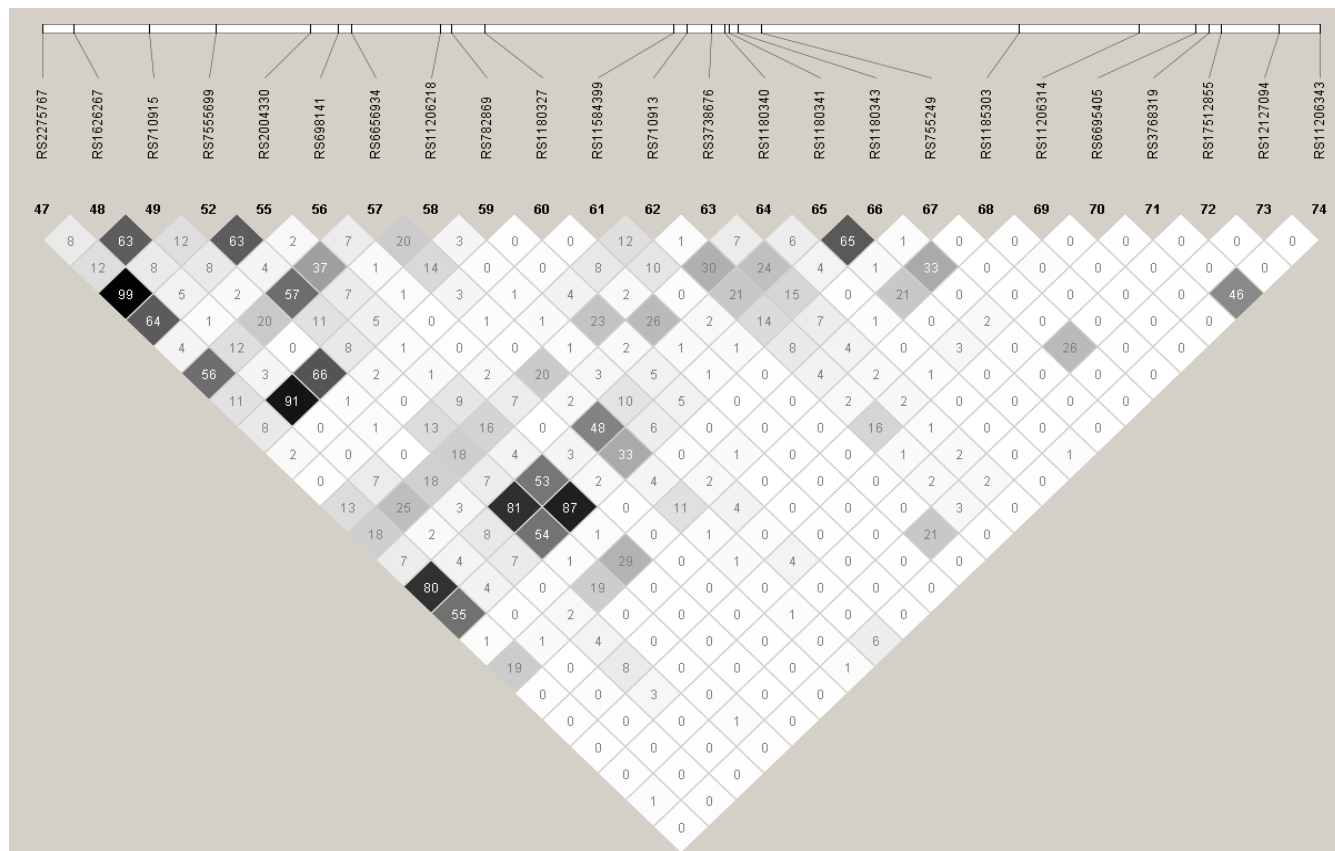

Supplement: S1 Fig — Plot of the linkage disequilibrium in both the EA (a) and AA (b) cohorts. We see different patterns of LD between the EA and AA cohorts for BMP8A. The European-Americans show a higher degree of LD than the African-Americans. Cells are colored according to the r2 with darker cells indicating a higher r2. (PDF) [file pone.0152670.s001.pdf]

S10 Fig

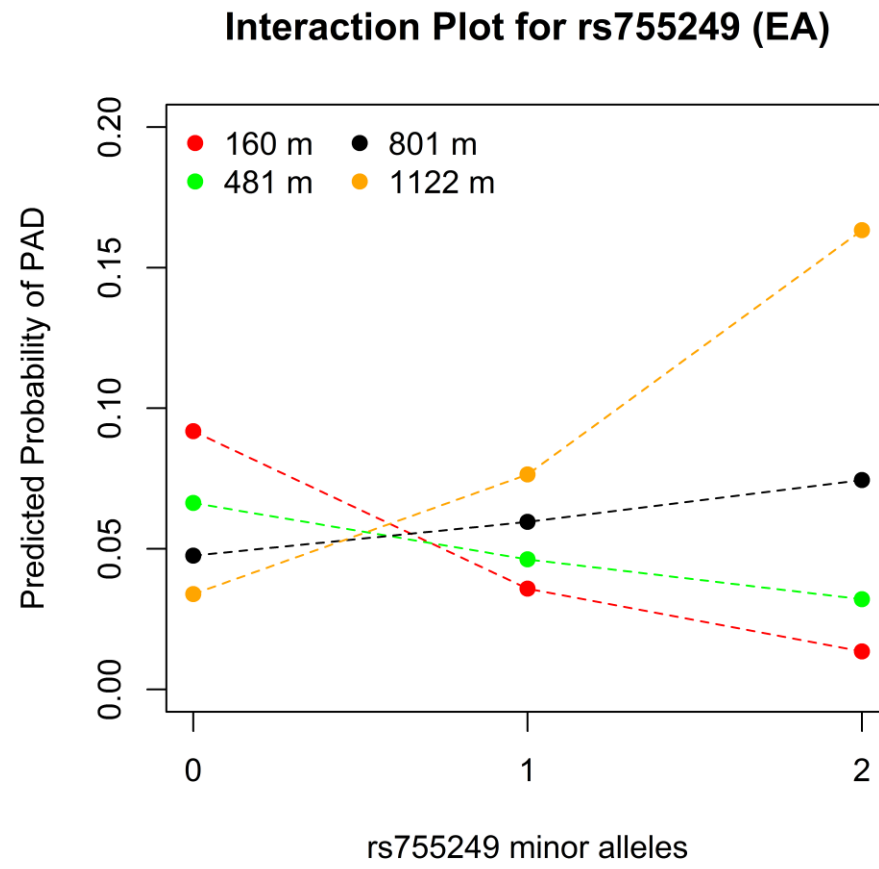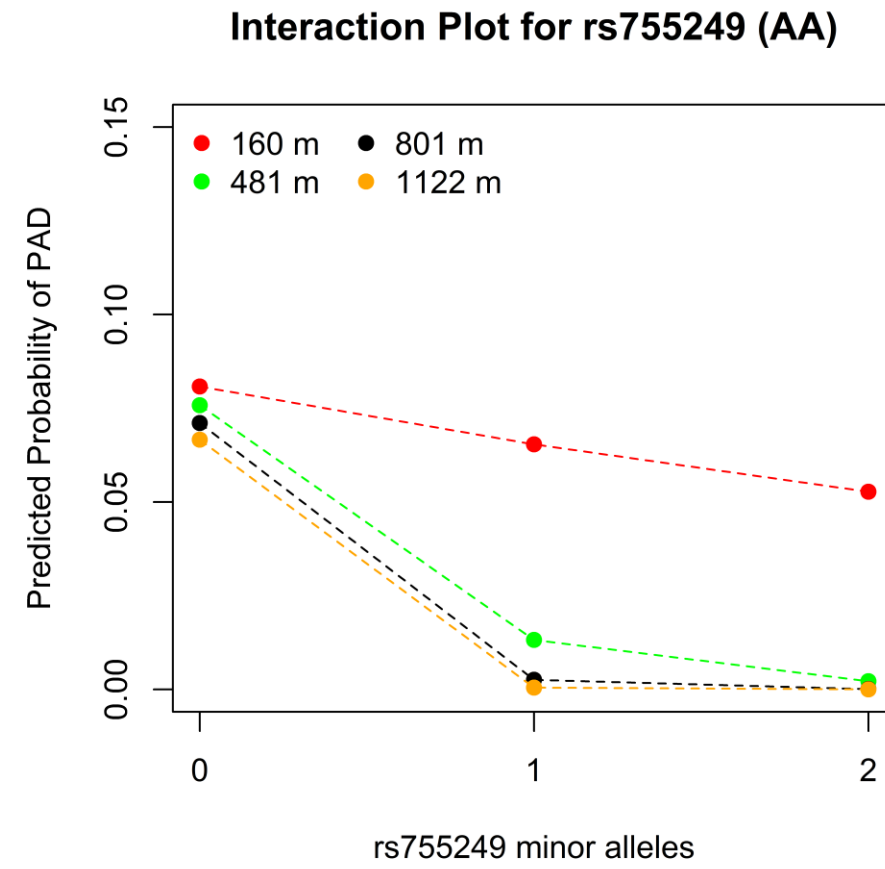

Supplement: S2 Fig — This figure shows the interaction between rs755249 and traffic exposure as associated with PAD in the EA and AA. On the x-axis is the number of minor alleles and on the y-axis is the predicted probability of PAD as given by our primary model. For the age term the average age in EA (61.2 y) and AA (56.3 y) was used and the sex was assumed to be male. For the principal components the average for each of the race-specific principal components was used. The colors correspond to the distance from primary residence to the nearest major roadway given in meters. (PDF) [file pone.0152670.s002.pdf]

S3 Fig

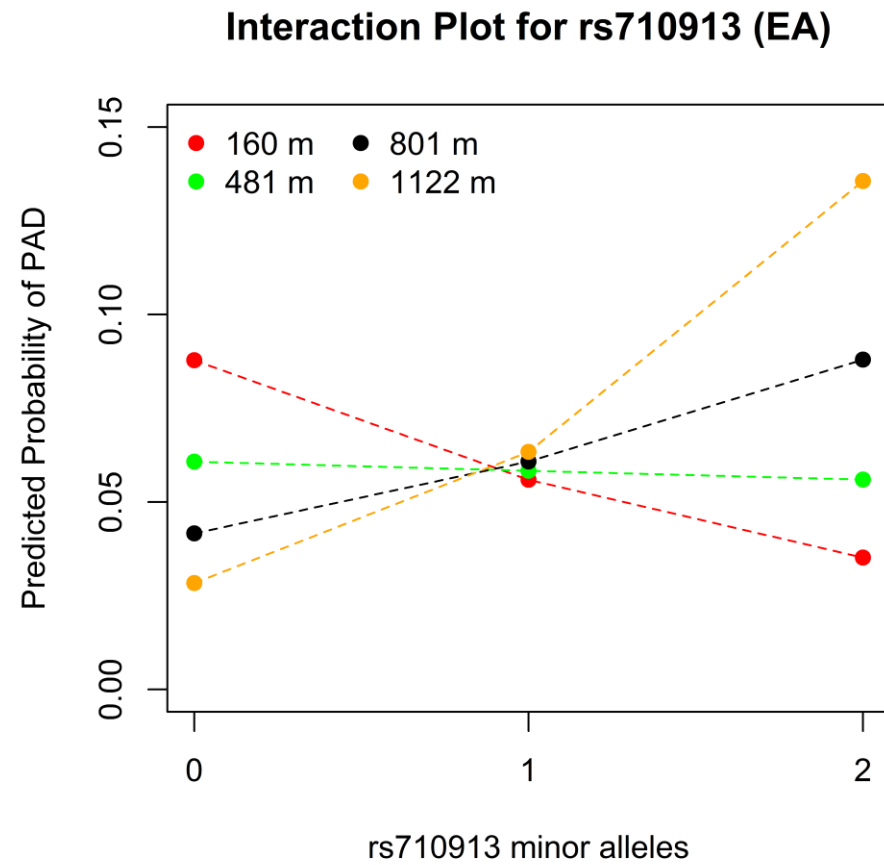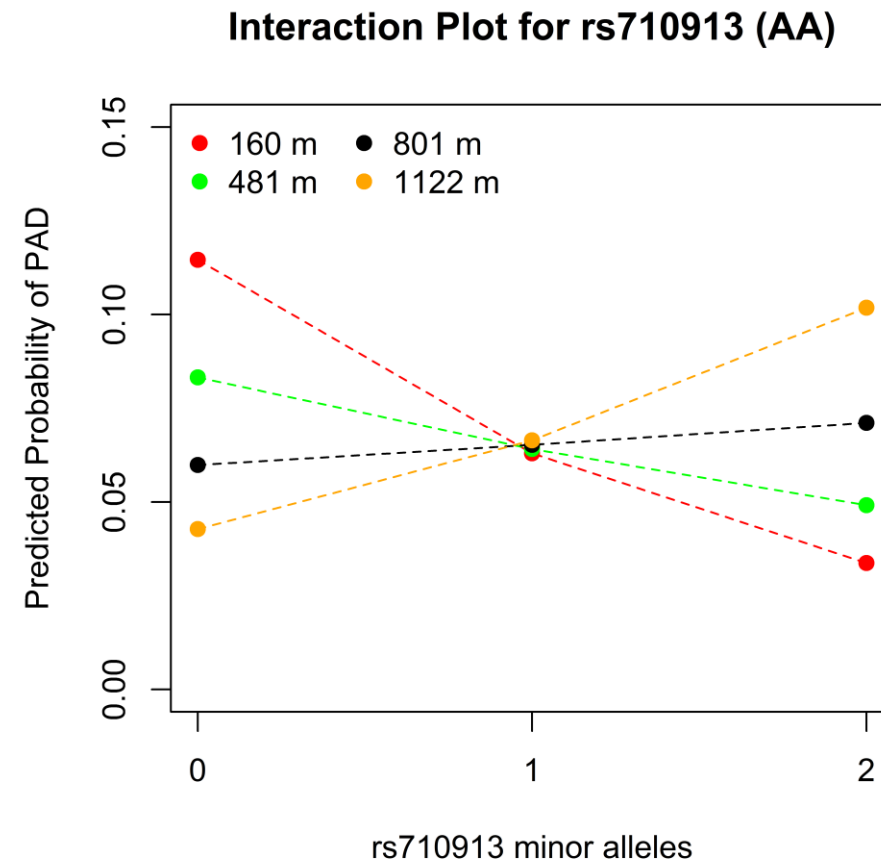

Supplement: S3 Fig — This figure shows the interaction between rs710913 and traffic exposure as associated with PAD in the EA and AA. On the x-axis is the number of minor alleles and on the y-axis is the predicted probability of PAD as given by our primary model. For the age term the average age in EA (61.2 y) and AA (56.3 y) was used and the sex was set to be male for both plots. For the principal components the race-specific average for each principal component was used. The colors correspond to the distance from primary residence to the nearest major roadway given in meters. We see a consistent effect for the interaction for both the EA and AA GWIS with the effect somewhat attenuated in the AA cohort which is consistent with the reduced p-value for the rs710913-traffic exposure interaction in this cohort. (PDF) [file pone.0152670.s003.pdf]
