# Supplementary material for: Genetic Variants in the Bone Morphogenic Protein Gene Family Modify the Association between Residential Exposure to Traffic and Peripheral Arterial Disease
Source: PLoS One. 2016 Apr 15;11(4):e0152670. doi: 10.1371/journal.pone.0152670 (PMC4833382; doi:10.1371/journal.pone.0152670)
Supplement: S1 Table — Clinical covariates for the CATHGEN cohort for the full cohort (a), the air pollution study cohort (b), and the genome-wide interaction study (GWIS) cohort (c). The GWIS cohort represents those CATHGEN participants used for this analysis. (PDF) [file pone.0152670.s004.pdf]

Supplemental Table 1: Clinical covariates separated by PAD status. P-values assessed via ANOVA for continuous covariates (Age and BMI), and via a Chi-squared test for all binary outcomes: Sex, Race, Smoking, Diabetes, Hypertension, Dyslipidemia.

Table 1				
1a. CATHGEN Clinical Covariates	All (N = 9334)	PAD cases (N = 709)	PAD Controls (N = 8625)	P
Age (SD)	60.8 (12)	65.5 (10.2)	60.4 (12.1)	< 0.001
BMI (SD)	30 (7.19)	29.1 (6.61)	30.1 (7.23)	< 0.001
Sex (% Female)	3531 (37.8)	225 (31.7)	3306 (38.3)	< 0.001
Race (% Caucasian)	6981 (74.8)	552 (77.9)	6429 (74.5)	< 0.001
Smoking (% Ever Smoke)	4439 (47.6)	503 (70.9)	3936 (45.6)	< 0.001
Diabetes (% Yes)	2640 (28.3)	294 (41.5)	2346 (27.2)	< 0.001
Hypertension (% Yes)	6277 (67.2)	574 (81)	5703 (66.1)	< 0.001
Dyslipidemia (% Yes)	5557 (59.5)	542 (76.4)	5015 (58.1)	< 0.001
1b. Air Pollution Study Cohort	All (N = 6066)	PAD cases (N = 478)	PAD Controls (N = 5588)	P
Age (SD)	61 (12)	64.7 (10.7)	60.7 (12.1)	< 0.001
BMI (SD)	30.1 (7.29)	29.3 (7.26)	30.2 (7.29)	0.010
Sex (% Female)	2333 (38.5)	139 (29.1)	2194 (39.3)	< 0.001
Race (% Caucasian)	4703 (77.5)	380 (79.5)	4323 (77.4)	0.31
Smoking (% Ever Smoke)	2946 (48.6)	352 (73.6)	2594 (46.4)	< 0.001
Diabetes (% Yes)	1749 (28.8)	196 (41)	1553 (27.8)	< 0.001
Hypertension (% Yes)	4150 (68.4)	398 (83.3)	3752 (67.1)	< 0.001
Dyslipidemia (% Yes)	3659 (60.3)	373 (78)	3286 (58.8)	< 0.001
1c. GWIS Cohort	All (N = 2177)	PAD cases (N = 138)	PAD Controls (N = 2039)	P
Age (SD)	60 (12)	65.2 (10.4)	59.6 (12)	< 0.001
BMI (SD)	30.4 (7.34)	29.9 (7.29)	30.4 (7.35)	0.41
Sex (% Female)	936 (43)	49 (35.5)	887 (43.5)	0.080
Race (% Caucasian)	1623 (74.6)	104 (75.4)	1519 (74.5)	0.90
Smoking (% Ever Smoke)	1051 (48.3)	97 (70.3)	954 (46.8)	< 0.001
Diabetes (% Yes)	665 (30.5)	65 (47.1)	600 (29.4)	< 0.001
Hypertension (% Yes)	1491 (68.5)	115 (83.3)	1376 (67.5)	< 0.001
Dyslipidemia (% Yes)	1286 (59.1)	109 (79)	1177 (57.7)	< 0.001
