# Supplementary material for: Genetic Variants in the Bone Morphogenic Protein Gene Family Modify the Association between Residential Exposure to Traffic and Peripheral Arterial Disease
Source: PLoS One. 2016 Apr 15;11(4):e0152670. doi: 10.1371/journal.pone.0152670 (PMC4833382; doi:10.1371/journal.pone.0152670)
Supplement: S2 Table — Results from the clinical factor adjusted model for variants with a P < 1x10^-4^ in this model. The model was adjusted for age, sex, BMI, hypertension, diabetes, smoking status, and hyperlipidemia. The model also included terms for the genetic main effect, environmental main effect, and SNP-traffic exposure interaction term. Results shown are those for the interaction term. (PDF) [file pone.0152670.s005.pdf]

Supplemental Table 2a. EA GWIS Full Model Results

SNP	Odds Ratio	SE	P
RS755249	3.27	0.24	2.86E-07
RS9409787	4.53	0.33	8.22E-07
RS17513135	3.23	0.25	9.06E-07
RS12651722	2.84	0.23	2.05E-06
RS10063408	2.90	0.23	2.12E-06
RS3738676	2.85	0.23	2.25E-06
RS6879255	2.81	0.23	2.56E-06
RS2431268	2.81	0.23	3.19E-06
RS16890431	9.20	0.62	3.28E-06
RS4904089	2.95	0.25	3.56E-06
RS2185458	2.95	0.25	3.69E-06
RS7539279	2.76	0.23	4.05E-06
RS2479587	7.22	0.49	4.25E-06
RS7520271	2.76	0.23	4.27E-06
RS2255486	495	2.91	4.79E-06
RS2653622	8.14	0.59	8.91E-06
RS1317681	3.35	0.30	1.10E-05
RS12195741	2.52	0.22	1.10E-05
RS10491083	5.18	0.45	1.14E-05
RS11206378	2.59	0.22	1.32E-05
RS9548897	2.69	0.23	1.41E-05
RS6873809	2.50	0.22	1.44E-05
RS2893113	7.97	0.55	1.48E-05
RS4660214	2.83	0.25	1.48E-05
RS2282231	2.82	0.25	1.55E-05
RS7448872	2.49	0.22	1.56E-05
RS16826069	2.80	0.25	1.56E-05
RS10050893	2.49	0.22	1.58E-05
RS2123811	2.49	0.22	1.60E-05
RS398293	0.37	0.23	1.62E-05
RS12883063	2.81	0.25	1.63E-05
RS2296173	2.82	0.25	1.66E-05
RS2296172	2.82	0.25	1.66E-05
RS3768302	2.82	0.25	1.66E-05
RS2746183	3.20	0.29	1.69E-05
RS12434963	0.38	0.23	1.73E-05
RS3899445	2.53	0.22	1.78E-05
RS16826093	2.81	0.25	1.79E-05
RS460911	2.73	0.24	1.88E-05
RS6594781	2.44	0.21	1.89E-05
RS9395234	0.28	0.31	2.00E-05
RS41290700	21182	11.16	2.03E-05
RS5992403	2.45	0.21	2.03E-05
RS6045173	0.41	0.21	2.07E-05
RS973148	2.60	0.23	2.28E-05
RS10437580	0.39	0.23	2.32E-05
RS10978620	2.77	0.25	2.33E-05
RS4340770	0.40	0.22	2.34E-05
RS12030495	2.48	0.22	2.53E-05
RS7863929	2.75	0.25	2.62E-05

RS11202287	4.10	0.36	2.67E-05
RS6780357	6.76	0.49	2.72E-05
RS4962067	4.12	0.37	2.73E-05
RS10983084	0.38	0.23	2.85E-05
RS11202282	3.59	0.32	2.91E-05
RS11717532	6.72	0.49	2.96E-05
RS11716183	6.72	0.49	2.96E-05
RS1411500	17.2	0.90	3.02E-05
RS17109801	2.76	0.25	3.06E-05
RS1867114	3.13	0.29	3.14E-05
RS1622879	0.41	0.22	3.18E-05
RS719793	0.36	0.25	3.18E-05
RS11966101	8.59	0.63	3.24E-05
RS7047726	3.98	0.36	3.30E-05
RS5746742	2.38	0.21	3.32E-05
RS6712892	0.26	0.34	3.36E-05
RS1180320	0.41	0.22	3.40E-05
RS6912824	5.53	0.49	3.48E-05
RS6570048	0.40	0.22	3.52E-05
RS7686844	2.34	0.21	3.71E-05
RS2255357	334	2.90	3.72E-05
RS6838749	2.34	0.21	3.80E-05
RS1555235	0.37	0.24	3.85E-05
RS1161240	0.42	0.22	4.10E-05
RS7747909	2.61	0.24	4.16E-05
RS2269722	0.43	0.21	4.19E-05
RS11733284	2.44	0.22	4.22E-05
RS3850947	2.85	0.26	4.22E-05
RS643099	3.79	0.36	4.28E-05
RS7780899	2.80	0.26	4.60E-05
RS7448169	2.35	0.21	4.62E-05
RS2129209	0.19	0.43	4.64E-05
RS4415146	2.46	0.22	4.69E-05
RS6893716	215	2.01	4.78E-05
RS10934751	0.39	0.23	4.98E-05
RS7554206	2.50	0.23	5.04E-05
RS10900675	2.41	0.22	5.06E-05
GA022530	2.63	0.25	5.34E-05
RS458975	2.56	0.24	5.39E-05
RS1610062	2.54	0.23	5.49E-05
RS710913	2.41	0.22	5.52E-05
RS6892152	2.39	0.22	5.54E-05
RS587404	2.48	0.23	5.59E-05
RS2283650	0.43	0.21	5.72E-05
RS10788933	2.53	0.24	5.72E-05
RS16890409	2.78	0.27	5.87E-05
RS12082516	0.30	0.31	5.87E-05
RS5992413	0.43	0.21	5.91E-05
RS927987	0.38	0.25	6.02E-05
RS1044176	5.58	0.46	6.02E-05
RS1029845	2.46	0.23	6.07E-05
RS681634	2.37	0.22	6.08E-05

RS4320303	2.32	0.21	6.18E-05
RS6594760	2.32	0.21	6.22E-05
RS2168002	2.32	0.21	6.22E-05
RS12623026	2.42	0.22	6.23E-05
RS16856197	13.7	0.78	6.29E-05
RS6440276	13.7	0.78	6.29E-05
RS2369754	2.32	0.21	6.31E-05
RS6894718	2.32	0.21	6.33E-05
RS3850905	2.37	0.22	6.36E-05
RS2275187	2.48	0.23	6.37E-05
RS1565078	2.42	0.22	6.42E-05
RS7576895	2.42	0.22	6.43E-05
RS6890382	2.37	0.22	6.45E-05
RS2275188	2.47	0.23	6.51E-05
RS6513310	3.76	0.39	6.66E-05
RS4134982	16.1	0.87	6.78E-05
RS7186248	0.40	0.23	6.80E-05
RS2732078	2.38	0.22	6.81E-05
RS13374459	2.30	0.21	6.92E-05
RS2866622	2.46	0.23	6.94E-05
RS10484879	2.55	0.24	6.98E-05
RS12552226	2.46	0.23	6.99E-05
RS4716100	0.39	0.24	7.11E-05
RS4736297	0.40	0.23	7.14E-05
RS2699502	0.41	0.23	7.52E-05
RS10493087	2.45	0.23	7.62E-05
RS1208217	0.41	0.23	7.68E-05
RS1384936	2.37	0.22	7.71E-05
RS10129421	2.46	0.24	7.75E-05
RS2111362	2.39	0.23	7.77E-05
RS17250154	0.24	0.38	7.79E-05
RS11803971	6.68	0.54	7.90E-05
RS8003722	3.10	0.30	7.92E-05
RS1080382	2.31	0.22	7.99E-05
RS15820	2.86	0.28	8.08E-05
RS1346901	2.29	0.21	8.10E-05
RS337520	2.61	0.25	8.25E-05
RS540599	2.26	0.21	8.33E-05
RS9466456	2.41	0.23	8.33E-05
RS7523716	0.37	0.26	8.40E-05
RS10741815	3.05	0.30	8.44E-05
RS16837533	2.90	0.28	8.60E-05
RS3118752	6.53	0.56	8.60E-05
RS1487393	2.39	0.22	8.62E-05
RS10788925	2.48	0.24	8.74E-05
RS913462	2.37	0.22	8.89E-05
RS321631	2.38	0.22	9.16E-05
RS11852841	2.28	0.21	9.21E-05
RS9546538	0.35	0.28	9.26E-05
RS9531002	2.40	0.23	9.38E-05
RS7933771	2.76	0.27	9.38E-05
RS2041944	0.43	0.22	9.41E-05

RS11794730	0.42	0.23	9.51E-05
RS1887696	2.31	0.22	9.54E-05
RS9593835	0.35	0.28	9.55E-05
RS1867115	3.18	0.31	9.57E-05
RS11806567	0.30	0.32	9.62E-05
RS540458	0.29	0.33	9.63E-05
RS4712500	4.05	0.39	9.78E-05
RS11249617	2.65	0.26	9.80E-05
RS12471388	2.67	0.27	9.84E-05
RS6726083	2.67	0.27	9.87E-05

Supplemental Table 2b. AA GWIS Full Model Results

PROBE	ODDS_RATIO	SE	SCORE_PVAL
RS9567406	58.4	2.40	1.44E-10
RS1638665	827	10.65	3.23E-10
RS13389599	23.2	1.49	4.25E-10
RS634138	6.51	0.71	3.18E-09
RS11039100	142	2.44	3.41E-09
RS3755899	4529	11.86	7.51E-09
RS7787478	9.38	0.67	1.57E-08
RS11190074	7.64	0.70	1.63E-08
RS6570628	26.9	0.86	2.25E-08
RS17147740	10.5	0.66	2.84E-08
RS9940555	7.57	0.54	3.39E-08
RS2989314	11.8	0.71	5.09E-08
RS750723	110	5.56	1.30E-07
RS7860288	20.3	0.79	1.55E-07
RS7022762	20.3	0.79	1.55E-07
RS1735894	6.64	0.54	2.61E-07
RS16965478	5.95	0.59	3.18E-07
RS16855732	23.4	1.10	4.21E-07
RS499832	6.21	0.49	4.77E-07
RS2161719	5.59	0.43	5.88E-07
RS8181880	27.5	1.04	6.00E-07
RS10979314	271	4.25	6.60E-07
RS11206019	37.0	1.42	6.75E-07
RS12335314	5.43	0.73	7.29E-07
RS9557207	27.4	1.04	7.30E-07
RS10507495	16.8	0.90	7.53E-07
RS17019537	8.57	0.80	9.80E-07
RS3181225	4.78	0.59	1.11E-06
RS28667979	21.5	0.81	1.11E-06
RS11220138	28.3	1.17	1.12E-06
RS4547347	124	2.32	1.15E-06
RS17080395	5.66	0.57	1.21E-06
RS11466653	119	5.56	1.25E-06
RS10039526	13.4	0.77	1.26E-06
RS11512640	27.6	1.17	1.31E-06
RS9824246	6.16	0.55	1.33E-06
RS11077983	4.97	0.48	1.38E-06
RS9397365	17.4	0.76	1.39E-06
RS11596966	67.0	1.48	1.40E-06
RS11942139	20.2	1.03	1.56E-06

RS2569254	4.70	0.59	1.65E-06
RS9747201	4.88	0.47	2.12E-06
RS242423	6.45	0.62	2.14E-06
RS9451860	69.9	2.18	2.17E-06
RS17037831	4.53	0.49	2.19E-06
RS9784649	15.4	0.76	2.26E-06
RS2073300	11.4	1.05	2.39E-06
RS3806708	663	7.09	2.46E-06
RS17130391	19.8	1.01	2.56E-06
RS8121203	25.1	1.07	2.57E-06
RS1539189	7.14	0.71	2.58E-06
RS13147122	26.2	1.27	2.97E-06
RS234284	16.7	0.79	3.33E-06
RS11815171	4.69	0.52	3.48E-06
RS1923894	11.3	0.66	3.56E-06
RS4239020	4.76	0.48	3.58E-06
RS17391705	61.3	1.51	3.59E-06
RS6713543	47.4	1.55	3.70E-06
RS10508774	12.0	0.71	3.72E-06
RS1912124	105	1.96	3.80E-06
RS9600550	34.2	1.13	4.22E-06
RS2391106	4.63	0.52	4.42E-06
RS10131663	4.37	0.41	4.42E-06
RS6057126	6.44	0.51	4.48E-06
RS13312727	24.5	1.17	4.64E-06
RS7046999	6.41	0.66	4.71E-06
RS1810494	42.9	4.51	4.73E-06
RS16832236	16.3	1.00	4.82E-06
RS660323	5.92	0.47	4.90E-06
RS4757882	5.83	0.54	4.91E-06
RS6698723	4.51	0.46	4.98E-06
RS11622263	290	5.74	5.24E-06
RS17143122	458	5.13	5.30E-06
RS10510755	729	6.99	5.30E-06
RS28382829	20.0	1.11	5.33E-06
RS16941207	12.4	0.69	5.34E-06
RS16892422	7.58	0.72	5.44E-06
RS6748245	36.8	1.30	5.79E-06
RS1392372	22.2	1.11	5.84E-06
RS6142859	67.0	1.73	5.85E-06
RS1786426	5.62	0.66	5.94E-06
RS962073	5.60	0.59	6.00E-06
RS4669572	5.47	0.51	6.02E-06
RS4919017	11.2	0.69	6.08E-06
RS10428206	24.0	1.27	6.30E-06
RS17121644	17.0	0.99	6.39E-06
RS2171475	20.9	0.95	6.52E-06
RS9596720	28.3	1.10	6.68E-06
RS1027896	17.8	1.15	6.72E-06
RS11819158	8.03	0.85	6.80E-06
RS10467515	28.5	1.10	6.80E-06
RS7605628	12.0	0.69	6.92E-06

RS919953	4.81	0.49	6.97E-06
RS10046574	13.6	0.88	7.02E-06
RS736088	61.0	1.73	7.38E-06
RS10002434	17.7	1.07	7.65E-06
RS10159109	31.1	1.41	7.84E-06
RS6980740	3.91	0.46	7.87E-06
RS12079836	4.41	0.45	8.15E-06
RS10484876	35.8	1.31	8.18E-06
RS10948567	26.6	1.25	8.49E-06
RS2056389	9.97	0.65	8.51E-06
RS13335729	4.43	0.41	8.62E-06
RS3787309	6.20	0.46	8.80E-06
RS12410212	31.9	1.42	8.89E-06
RS17307778	14.9	1.05	9.07E-06
RS4596377	34.9	1.36	9.12E-06
RS497022	11.0	0.67	9.14E-06
RS3858306	4.90	0.44	9.69E-06
RS16962242	17.0	0.86	9.74E-06
RS7339053	13.0	0.72	9.83E-06
RS720844	3.98	0.39	1.00E-05
RS7905504	8.73	0.79	1.02E-05
RS12605132	19.3	1.02	1.03E-05
RS12257301	5.45	0.46	1.06E-05
RS615026	16.7	1.11	1.09E-05
RS17836804	10.8	0.66	1.10E-05
RS10819779	43.8	1.62	1.13E-05
RS1053726	11.4	0.71	1.14E-05
RS4134872	15.9	0.83	1.14E-05
RS17043519	13.2	0.82	1.19E-05
RS16914101	4.81	0.46	1.19E-05
RS952932	19.3	1.04	1.20E-05
RS7200988	13.2	0.73	1.25E-05
RS11209878	5.69	0.76	1.28E-05
RS4414501	4.04	0.37	1.28E-05
RS7195194	4.04	0.37	1.28E-05
RS17064440	5.45	0.64	1.29E-05
RS6957127	11.4	0.85	1.30E-05
RS7315359	4.29	0.42	1.30E-05
RS10021155	18.2	1.09	1.33E-05
RS10129758	5.21	0.50	1.33E-05
RS7543809	9.17	0.61	1.34E-05
RS16832352	18.0	1.09	1.34E-05
RS2510157	3.98	0.45	1.35E-05
RS256811	3.61	0.39	1.35E-05
RS16944118	5.45	0.50	1.37E-05
RS7970417	8.73	0.58	1.40E-05
RS17149626	18.2	1.09	1.40E-05
RS35486143	50.7	5.30	1.41E-05
RS16919174	113	2.36	1.43E-05
RS4837752	3.86	0.40	1.43E-05
RS10190087	8.97	0.61	1.45E-05
RS13426621	19.7	1.10	1.45E-05

RS12541465	4.26	0.44	1.46E-05
RS9477218	44.6	1.35	1.47E-05
RS17853024	15.5	0.75	1.48E-05
RS3181718	15.4	0.88	1.51E-05
RS4789729	3.96	0.40	1.53E-05
RS9567063	14.0	0.82	1.53E-05
RS9554568	15.1	0.87	1.55E-05
RS9633677	11.8	0.73	1.58E-05
RS7980687	4.73	0.43	1.59E-05
RS1846413	4.61	0.55	1.59E-05
RS6254	20.2	1.03	1.59E-05
RS17204629	14.5	1.01	1.60E-05
RS11912074	3.71	0.37	1.63E-05
RS10018408	60.9	1.91	1.63E-05
RS9261219	12.6	0.80	1.65E-05
RS12166034	7.97	0.64	1.66E-05
RS1147572	27.3	1.11	1.67E-05
RS1593415	14.5	1.05	1.68E-05
RS12777098	16.6	1.02	1.70E-05
RS12445943	5.43	0.46	1.73E-05
RS11012350	4.63	0.53	1.73E-05
RS1881466	20.5	1.15	1.74E-05
RS12056645	4.82	0.46	1.76E-05
RS9464413	10.0	0.63	1.76E-05
RS1796306	11.2	0.75	1.76E-05
RS9396245	9.97	0.63	1.76E-05
RS7803922	4.43	0.42	1.77E-05
RS4943266	5.71	0.61	1.78E-05
RS7768403	52.9	1.88	1.78E-05
RS12807369	5.37	0.50	1.79E-05
RS2516698	4.37	0.47	1.80E-05
RS11773898	6.07	0.53	1.83E-05
RS7295749	4.04	0.52	1.83E-05
RS7989565	75.5	1.70	1.86E-05
RS2592394	4.87	0.44	1.88E-05
RS9304301	17.3	0.98	1.89E-05
RS7301563	12.3	0.92	1.91E-05
RS12612150	10.6	0.79	1.91E-05
RS10508336	34.8	1.42	1.92E-05
RS17168377	10.4	0.68	1.93E-05
RS13312723	20.2	1.15	1.93E-05
RS8064257	9.17	0.66	1.93E-05
RS16975650	8.24	0.58	1.93E-05
RS17047453	9.87	0.64	1.99E-05
RS9557176	14.8	0.87	2.00E-05
RS2243411	9.39	0.60	2.01E-05
RS1684978	3.70	0.38	2.01E-05
RS8030348	16.0	0.98	2.01E-05
RS1960306	4.59	0.54	2.04E-05
RS2243796	13.4	0.93	2.04E-05
RS16981522	905	6.73	2.08E-05
RS12024301	4.63	0.58	2.08E-05

RS7901466	11.9	0.71	2.10E-05
RS3784246	12.4	0.77	2.10E-05
RS7655674	7.83	0.56	2.11E-05
RS11975647	4.97	0.55	2.15E-05
RS10115371	5.54	0.45	2.19E-05
RS6909762	50.8	1.73	2.19E-05
RS12038357	36.0	1.20	2.20E-05
RS11771793	4.39	0.41	2.21E-05
RS1451882	13.1	0.91	2.22E-05
RS7730356	14.1	0.88	2.23E-05
RS16976904	16.3	0.88	2.25E-05
RS1013870	19.4	1.15	2.26E-05
RS9345068	12.3	0.84	2.26E-05
RS10178114	20.0	1.52	2.28E-05
RS7323678	4.55	0.50	2.29E-05
RS16910400	4.95	0.56	2.30E-05
RS8073525	13.7	0.91	2.32E-05
RS4605886	5.20	0.56	2.35E-05
RS12247913	3.86	0.50	2.37E-05
RS12175863	19.3	1.31	2.40E-05
RS1372202	10.7	0.72	2.40E-05
RS10978077	4.58	0.45	2.41E-05
RS7724962	3.79	0.44	2.41E-05
RS11618570	4.01	0.40	2.44E-05
RS2099269	8.46	0.55	2.47E-05
RS17123327	12.6	0.90	2.50E-05
RS17135409	3.70	0.45	2.51E-05
RS16904171	17.4	1.05	2.54E-05
RS17050725	12.4	1.03	2.54E-05
RS12306432	9.06	0.61	2.57E-05
RS7386256	7.62	0.65	2.64E-05
RS17806552	23.6	1.21	2.65E-05
RS7830816	4.25	0.42	2.67E-05
RS2048917	4.31	0.41	2.67E-05
RS16965676	23.4	1.00	2.67E-05
RS901720	6.22	0.51	2.68E-05
RS12214948	12.3	0.91	2.68E-05
RS1342760	20.1	1.00	2.71E-05
RS10138933	4.63	0.51	2.73E-05
RS7874051	26144	9.15	2.74E-05
RS10134584	4.99	0.51	2.74E-05
RS17139138	6.97	0.51	2.75E-05
RS17248859	6.13	0.62	2.76E-05
RS12640471	22.4	1.42	2.78E-05
RS12308044	7.40	0.55	2.78E-05
RS17039638	18.4	1.08	2.80E-05
RS4134901	30.0	1.55	2.82E-05
RS10947929	4.19	0.41	2.87E-05
RS12047483	11.2	0.77	2.90E-05
RS239027	3.65	0.38	2.93E-05
RS4752066	4.25	0.48	2.96E-05
RS8973	5.74	0.64	2.97E-05

RS6697084	9.33	0.66	3.00E-05
RS623899	46.1	1.67	3.01E-05
RS188750	7.83	0.60	3.07E-05
RS4831658	4.69	0.50	3.08E-05
RS13259685	4.17	0.38	3.09E-05
RS11874308	3.78	0.39	3.13E-05
RS11990843	4.59	0.48	3.13E-05
RS9481643	14.4	0.89	3.17E-05
RS10999594	3.87	0.48	3.19E-05
RS1503849	6.83	0.53	3.22E-05
RS2281558	4.10	0.40	3.23E-05
RS12582044	8.73	0.66	3.25E-05
RS665974	4.93	0.45	3.30E-05
RS1055970	4.28	0.49	3.35E-05
RS936115	17.5	0.95	3.46E-05
RS1378683	17.5	0.95	3.46E-05
RS12769955	13.5	0.75	3.48E-05
RS17058330	4.71	0.53	3.48E-05
RS12056981	56.8	1.83	3.49E-05
RS6464215	4.67	0.42	3.51E-05
RS17064584	5.28	0.60	3.57E-05
RS12528404	8.65	0.70	3.58E-05
RS1112868	38.2	1.28	3.60E-05
RS11605275	5.22	0.65	3.64E-05
RS2066098	92.4	2.80	3.70E-05
RS10827150	12.1	0.82	3.75E-05
RS16869245	14.3	0.95	3.75E-05
RS1550558	4.85	0.45	3.77E-05
RS12196677	12.4	0.75	3.77E-05
RS13257525	3.92	0.37	3.77E-05
RS17725600	13.2	0.91	3.78E-05
RS939052	3.74	0.39	3.79E-05
RS2033466	10.6	0.68	3.81E-05
RS13141109	4.25	0.48	3.81E-05
RS6537319	13.1	0.91	3.84E-05
RS6677436	4.88	0.41	3.84E-05
RS10170657	8.88	0.71	3.86E-05
RS7752599	3.97	0.43	3.92E-05
RS10518850	15.7	0.88	3.98E-05
RS16976913	15.7	0.88	3.98E-05
RS12247544	4.50	0.49	4.01E-05
RS7358822	4.01	0.46	4.04E-05
RS16852893	6.35	0.64	4.04E-05
RS328996	9.07	0.63	4.05E-05
RS12704294	11272	10.59	4.05E-05
RS16953011	19.4	1.04	4.12E-05
RS10086104	10.5	0.67	4.12E-05
RS17194824	4.12	0.46	4.13E-05
RS10487085	3.72	0.40	4.13E-05
RS10408321	6.76	0.68	4.14E-05
RS13275996	80.7	2.34	4.15E-05
RS12458041	9.84	0.65	4.23E-05

RS2903016	9.09	0.61	4.24E-05
RS1048664	4.12	0.46	4.27E-05
RS6678735	5.31	0.71	4.28E-05
RS4948722	12.8	0.86	4.29E-05
RS2844754	4.11	0.46	4.35E-05
RS9365409	202	3.76	4.36E-05
RS17081017	12.0	0.78	4.38E-05
RS9853287	10.6	0.75	4.39E-05
RS16903467	15.9	1.01	4.39E-05
RS9356334	67.0	2.13	4.40E-05
RS12980609	3.48	0.40	4.40E-05
RS10001567	10.3	0.73	4.45E-05
RS9833934	10.8	0.78	4.45E-05
RS17469385	9.95	0.98	4.47E-05
RS10059433	4.78	0.57	4.47E-05
RS11915226	5.05	0.65	4.48E-05
RS1450667	347	6.20	4.50E-05
RS9394969	5.38	0.44	4.54E-05
RS9367486	38.6	1.41	4.56E-05
RS720840	10.2	0.69	4.60E-05
RS10130219	3.82	0.53	4.60E-05
RS973954	3.89	0.45	4.62E-05
RS4798730	2059	4.01	4.66E-05
RS7563103	5.03	0.47	4.66E-05
RS582581	49.1	1.69	4.67E-05
RS9859653	8.69	0.66	4.70E-05
RS2389672	4.41	0.48	4.72E-05
RS11109007	17.0	1.09	4.75E-05
RS6830548	4.30	0.53	4.79E-05
RS7644677	10.1	0.83	4.80E-05
RS10196592	34.7	1.60	4.80E-05
RS17007551	8.02	0.61	4.81E-05
RS3219474	7.94	0.77	4.84E-05
RS2044457	11.0	0.72	4.86E-05
RS7821128	3.81	0.41	4.87E-05
RS12574588	34.8	1.38	4.87E-05
RS283619	4.08	0.38	4.88E-05
RS35918317	14.6	0.85	4.88E-05
RS11022114	11.1	0.73	4.90E-05
RS10106404	10.6	0.76	4.92E-05
RS17396885	7.31	0.63	4.92E-05
RS9365411	4.45	0.46	4.93E-05
GA009992	4.20	0.44	4.93E-05
RS11827555	4.41	0.57	4.94E-05
RS12108835	21.1	1.13	4.95E-05
GA010364	4.30	0.43	4.96E-05
RS895423	11.8	0.76	5.00E-05
RS9866608	7.01	0.54	5.01E-05
RS3905000	4.42	0.45	5.01E-05
RS6564716	4.36	0.51	5.07E-05
RS17025036	4.18	0.49	5.08E-05
RS4714513	4.30	0.40	5.09E-05

RS6913042	10.5	0.66	5.09E-05
RS17153639	8.09	0.63	5.12E-05
RS6127146	20.0	1.10	5.12E-05
RS7185036	3.80	0.38	5.15E-05
RS672095	9.49	0.66	5.19E-05
RS10023523	4.20	0.40	5.19E-05
RS3774902	3.84	0.47	5.21E-05
RS11853488	9.30	0.86	5.23E-05
RS12915512	9.49	0.68	5.25E-05
RS28628372	9.50	0.68	5.26E-05
RS6873939	4.11	0.45	5.27E-05
RS10252263	4.73	0.42	5.28E-05
RS8892	3.97	0.44	5.31E-05
RS7982202	6.24	0.72	5.32E-05
RS2382882	4.24	0.50	5.37E-05
RS9468600	10.7	0.84	5.42E-05
RS6725219	15.2	1.08	5.43E-05
RS4134885	25.0	1.52	5.45E-05
RS9266777	48.6	1.90	5.45E-05
RS17429538	28.7	1.20	5.50E-05
RS10893404	4.28	0.43	5.53E-05
RS16923623	9.35	0.64	5.56E-05
RS17125553	4.43	0.47	5.56E-05
RS17405840	21.3	1.20	5.57E-05
RS749822	17.3	1.27	5.58E-05
RS720214	6.59	0.54	5.60E-05
RS11997816	10.1	0.96	5.66E-05
RS711825	4.39	0.42	5.66E-05
RS4867495	12.3	1.00	5.68E-05
RS9872679	6.59	0.54	5.75E-05
RS7902671	12.9	0.85	5.80E-05
RS575323	9.81	0.73	5.82E-05
RS12906810	10.2	0.68	5.88E-05
RS202390	10.4	0.77	5.88E-05
RS510463	9.80	0.73	5.89E-05
RS1540151	5.07	0.44	5.90E-05
RS1671400	6.84	0.56	5.92E-05
RS9448502	23.6	1.20	6.02E-05
RS9382099	27.7	1.52	6.02E-05
RS2280569	4.41	0.41	6.02E-05
RS225872	19.9	1.23	6.06E-05
RS17047440	7.76	0.60	6.10E-05
RS28496502	3.97	0.39	6.13E-05
RS693978	4.70	0.42	6.13E-05
RS9822767	4.17	0.47	6.18E-05
RS7677523	10.4	0.78	6.19E-05
RS34509814	9.47	0.69	6.24E-05
RS9484096	12.8	1.06	6.25E-05
RS12232326	17.6	0.96	6.28E-05
RS4771953	10.6	0.69	6.29E-05
RS11646066	4.90	0.44	6.53E-05
RS9890685	11.9	0.86	6.56E-05

RS16837982	17.7	1.03	6.58E-05
RS16891597	4.26	0.47	6.59E-05
RS4721648	0.19	0.46	6.60E-05
RS8133734	7.85	0.65	6.60E-05
RS3737269	12.0	0.77	6.62E-05
RS10094712	9.83	0.80	6.63E-05
RS28539472	3.95	0.39	6.63E-05
RS3123501	8.84	0.62	6.65E-05
RS907795	11.7	0.72	6.67E-05
RS716478	76.3	1.98	6.68E-05
RS4634736	8.84	0.62	6.74E-05
RS7088089	3.52	0.48	6.79E-05
RS7350409	12.1	0.79	6.79E-05
RS16856762	2454	6.66	6.87E-05
RS1028120	3.46	0.37	6.88E-05
RS2014220	4.78	0.42	6.89E-05
RS12572135	12.1	0.79	6.90E-05
RS6480463	4.50	0.40	6.99E-05
RS6955966	4.72	0.42	6.99E-05
RS7185877	3.94	0.39	7.00E-05
RS6804377	4.19	0.43	7.04E-05
RS7754365	11.3	0.83	7.09E-05
RS12059484	4.17	0.45	7.09E-05
RS4629304	4.20	0.50	7.12E-05
RS6113950	12.8	0.85	7.19E-05
RS34717587	27.3	1.53	7.20E-05
RS3125785	8.72	0.62	7.26E-05
RS4945880	9.65	0.72	7.32E-05
RS1396531	9.24	0.84	7.37E-05
RS328489	9.46	0.76	7.38E-05
RS9328707	4.00	0.39	7.40E-05
RS3915964	47.2	1.90	7.43E-05
RS4129826	3.81	0.37	7.44E-05
RS2798815	3.66	0.42	7.51E-05
RS3885071	7.56	0.61	7.52E-05
RS1511576	5.75	0.47	7.53E-05
RS10462823	12.8	1.02	7.61E-05
RS9821348	14.5	0.97	7.62E-05
RS1488831	8.16	0.59	7.64E-05
RS39986	3.20	0.37	7.65E-05
RS3088324	10.4	0.73	7.69E-05
RS35142427	4.17	0.44	7.71E-05
RS10218979	12.5	0.87	7.73E-05
RS9304276	4.31	0.52	7.76E-05
RS165569	3.83	0.43	7.80E-05
RS8020981	12.0	1.10	7.81E-05
RS4686334	7.71	0.60	7.82E-05
RS12422977	14.8	0.94	7.85E-05
RS16965811	4.68	0.63	7.86E-05
RS561735	9.63	0.73	7.89E-05
RS7261777	4.34	0.44	7.96E-05
RS7861556	4.01	0.42	7.97E-05

RS17194551	4.78	0.62	7.97E-05
RS225475	3.65	0.45	8.04E-05
RS7873053	4.24	0.40	8.04E-05
RS9867900	3.99	0.43	8.06E-05
RS7974564	6.49	0.52	8.14E-05
RS4368817	12.2	0.89	8.17E-05
RS939048	3.50	0.38	8.20E-05
RS6880907	4.29	0.55	8.27E-05
RS10173820	4.19	0.43	8.29E-05
RS1203814	3.95	0.38	8.32E-05
RS1527087	3.98	0.50	8.35E-05
RS163253	7.65	0.62	8.35E-05
RS17006368	25.8	1.21	8.36E-05
RS7337274	10.2	0.73	8.39E-05
RS958888	10.6	0.72	8.40E-05
RS4684618	6.46	0.58	8.42E-05
RS17081086	11.2	0.76	8.43E-05
RS7645471	4.42	0.40	8.45E-05
RS10117316	24.3	1.41	8.48E-05
RS10236400	3.90	0.45	8.52E-05
RS12354	14.9	1.09	8.53E-05
RS12419635	17.9	1.05	8.53E-05
RS16857235	6.25	0.57	8.65E-05
RS1563066	4.81	0.44	8.66E-05
RS2843557	27.0	1.37	8.72E-05
RS17065076	6.34	0.55	8.74E-05
RS10181159	15.5	1.07	8.75E-05
RS17138263	4.09	0.49	8.81E-05
RS12061545	4.52	0.45	8.85E-05
RS6982045	10.8	0.77	8.89E-05
RS16919776	10.1	0.82	8.90E-05
RS6789607	4.98	0.43	8.97E-05
RS519800	3.72	0.50	9.00E-05
RS28588913	9.39	0.67	9.03E-05
RS36046152	11.9	0.92	9.06E-05
RS405031	4.30	0.41	9.08E-05
RS11602119	26.3	1.61	9.08E-05
RS10421078	21.9	1.09	9.09E-05
RS4847100	5.79	0.46	9.09E-05
RS11466659	7.76	0.65	9.10E-05
RS11466648	7.76	0.65	9.10E-05
RS17473999	11.1	0.74	9.14E-05
RS3860270	14.2	0.86	9.15E-05
RS16868737	7.07	0.58	9.24E-05
RS4910297	4.38	0.40	9.25E-05
RS131992	4.26	0.43	9.26E-05
RS12813784	4.20	0.48	9.27E-05
RS11971803	4.77	0.53	9.34E-05
RS8083849	4.23	0.39	9.35E-05
RS6420969	4.12	0.56	9.40E-05
RS13395146	7.15	0.58	9.49E-05
RS13433067	7.15	0.58	9.49E-05

RS16983864	7.15	0.58	9.49E-05
RS10166369	7.15	0.58	9.49E-05
RS10177628	7.15	0.58	9.49E-05
RS9306857	7.15	0.58	9.49E-05
RS10031329	9.55	0.65	9.51E-05
RS12268694	20.9	1.34	9.53E-05
RS17148760	12.2	0.92	9.54E-05
RS590503	10.3	0.82	9.55E-05
RS13390998	16.5	1.44	9.62E-05
RS12101957	9.26	0.66	9.64E-05
RS28394527	9.26	0.66	9.64E-05
RS11183471	6.96	0.57	9.68E-05
RS11851536	3.65	0.50	9.77E-05
RS8068326	11.4	0.86	9.78E-05
RS7995134	4.55	0.57	9.81E-05
RS4897936	15.6	1.21	9.87E-05
RS1055160	3.92	0.42	9.91E-05
RS2622695	5.10	0.43	9.93E-05
RS1250309	8.88	0.64	9.96E-05
RS16938445	8.15	0.66	9.97E-05
RS11222977	4.33	0.43	9.97E-05
