# Supplementary material for: Genetic Variants in the Bone Morphogenic Protein Gene Family Modify the Association between Residential Exposure to Traffic and Peripheral Arterial Disease
Source: PLoS One. 2016 Apr 15;11(4):e0152670. doi: 10.1371/journal.pone.0152670 (PMC4833382; doi:10.1371/journal.pone.0152670)
Supplement: S3 Table — Results from the primary GWIS model which adjusted for age, sex, and race-specific principal components to remove population substructure for all interactions with a P < 1x10^-4^. In addition to age and sex, the model also included terms for the genetic main effect, environmental main effect, and SNP-traffic exposure interaction term. Results shown are those for the interaction term. (PDF) [file pone.0152670.s006.pdf]

S3 Table (a). EA GWIS Associations with $P < 1x10^{-4}$ in primary model (race, sex, and principal component adjusted)

SNP	Odds Ratio	SE	P
rs755249	3.45	0.23	2.29E-08
rs9409787	4.93	0.33	6.87E-08
rs17513135	3.34	0.24	1.33E-07
rs16890431	10.29	0.62	7.40E-07
rs4660214	3.08	0.24	1.26E-06
rs2296172	3.08	0.24	1.28E-06
rs3768302	3.08	0.24	1.28E-06
rs2296173	3.08	0.24	1.28E-06
rs16826093	3.07	0.24	1.33E-06
rs16826069	2.99	0.24	1.35E-06
rs7539279	2.81	0.22	1.41E-06
rs3738676	2.81	0.22	1.51E-06
rs7520271	2.80	0.22	1.55E-06
rs2282231	3.07	0.25	1.62E-06
rs1317681	3.57	0.29	2.21E-06
rs2479587	7.07	0.48	2.26E-06
rs10063408	2.83	0.23	3.57E-06
rs10491083	5.27	0.45	4.51E-06
rs11206378	2.64	0.22	5.98E-06
rs6879255	2.68	0.22	6.06E-06
rs12195741	2.50	0.21	6.32E-06
rs9548897	2.76	0.23	6.61E-06
rs12651722	2.66	0.23	7.36E-06
rs6045173	0.39	0.21	7.70E-06
rs10788933	2.76	0.23	7.72E-06
rs2431268	2.67	0.23	7.76E-06
rs2653622	7.67	0.57	7.93E-06
rs11202287	4.31	0.36	8.55E-06
rs10788925	2.71	0.23	1.08E-05
rs12434963	0.39	0.22	1.32E-05
rs10979826	3.32	0.28	1.45E-05
rs11202282	3.69	0.32	1.48E-05
rs3850947	2.89	0.25	1.58E-05
rs16837533	3.19	0.28	1.59E-05
rs587404	2.57	0.22	1.69E-05
rs643099	3.92	0.37	1.69E-05
rs11249617	2.73	0.25	1.77E-05
rs2275188	2.56	0.22	1.79E-05
rs2275187	2.57	0.22	1.80E-05
rs7554206	2.56	0.22	1.82E-05
rs4904089	2.72	0.25	1.86E-05
rs2484749	2.65	0.23	1.92E-05
rs13374459	2.47	0.21	1.97E-05
rs2185458	2.70	0.25	1.98E-05
rs460911	2.67	0.24	2.03E-05
rs2746183	3.09	0.28	2.16E-05
rs7186248	0.39	0.23	2.18E-05
rs17109801	2.65	0.24	2.22E-05
rs6873809	2.39	0.21	2.24E-05
rs16890409	2.85	0.26	2.24E-05
rs2123811	2.39	0.21	2.37E-05
rs10050893	2.39	0.21	2.37E-05
rs10493087	2.53	0.22	2.39E-05
rs7448872	2.38	0.21	2.40E-05
rs398293	0.39	0.23	2.54E-05

rs11966101	9.26	0.65	2.62E-05
rs6893716	103.25	1.72	2.63E-05
rs1622879	0.41	0.21	2.80E-05
rs4962067	3.95	0.36	2.87E-05
rs12030495	2.39	0.21	2.91E-05
rs2866622	2.54	0.23	2.95E-05
rs6780357	5.91	0.46	2.96E-05
rs710913	2.42	0.21	3.04E-05
rs7780899	2.81	0.26	3.15E-05
rs6594781	2.33	0.21	3.19E-05
rs4340770	0.41	0.21	3.20E-05
rs1867114	3.04	0.29	3.20E-05
rs1537818	2.51	0.23	3.21E-05
rs11903500	2.49	0.23	3.22E-05
rs2129209	0.19	0.42	3.60E-05
rs1180320	0.42	0.21	3.60E-05
rs11716183	5.83	0.46	3.65E-05
rs11717532	5.83	0.46	3.65E-05
rs6712892	0.26	0.34	3.78E-05
rs2699502	0.39	0.23	3.88E-05
rs1208217	0.40	0.23	4.32E-05
rs11733284	2.38	0.22	4.57E-05
rs6132020	2.48	0.23	4.59E-05
rs1161240	0.43	0.21	4.71E-05
rs973148	2.47	0.23	4.77E-05
rs1411500	14.78	0.87	4.81E-05
rs10983084	0.40	0.23	4.89E-05
rs12574080	4.90	0.43	4.99E-05
rs7176855	3.55	0.34	5.00E-05
rs10978620	2.62	0.25	5.01E-05
rs1080382	2.33	0.21	5.02E-05
rs7863929	2.61	0.24	5.19E-05
rs16856197	11.52	0.73	5.30E-05
rs6440276	11.52	0.73	5.31E-05
rs337520	2.63	0.24	5.49E-05
rs10437580	0.41	0.22	5.61E-05
rs1610062	2.49	0.23	5.63E-05
rs6570048	0.42	0.22	5.80E-05
rs4716100	0.38	0.24	5.91E-05
rs9466456	2.44	0.23	5.95E-05
rs11852841	2.30	0.21	5.96E-05
rs16975251	2.46	0.23	5.96E-05
rs12471388	2.69	0.27	5.97E-05
rs6726083	2.70	0.27	5.99E-05
rs16981576	4.53	0.40	6.01E-05
rs9395234	0.30	0.31	6.02E-05
rs12883063	2.53	0.24	6.09E-05
rs41275646	7.64	0.62	6.14E-05
rs10934751	0.40	0.23	6.15E-05
rs7448169	2.26	0.21	6.42E-05
rs7047726	3.67	0.35	6.43E-05
rs321631	2.36	0.22	6.45E-05
rs7031088	2.38	0.22	6.85E-05
rs1510937	0.40	0.23	6.92E-05
rs2294845	2.36	0.22	6.92E-05
rs1345517	2.68	0.26	6.97E-05
rs6894718	2.26	0.21	7.00E-05

rs16936269	21.21	1.04	7.07E-05
rs16936267	21.21	1.04	7.07E-05
rs11848	2.98	0.30	7.12E-05
rs10821352	6.66	0.55	7.23E-05
rs6594760	2.25	0.21	7.32E-05
rs2168002	2.25	0.21	7.32E-05
rs3803071	0.43	0.21	7.38E-05
rs6786606	2.73	0.26	7.42E-05
rs4320303	2.25	0.21	7.43E-05
rs7555699	2.31	0.21	7.45E-05
rs2275767	2.32	0.22	7.49E-05
rs2369754	2.25	0.21	7.50E-05
rs4712500	4.00	0.38	7.50E-05
rs2987202	5.93	0.52	7.85E-05
rs11154722	3.27	0.32	7.90E-05
rs5011374	2.39	0.22	7.91E-05
rs12623026	2.37	0.22	8.07E-05
rs9531002	2.37	0.22	8.16E-05
rs1867115	3.17	0.31	8.25E-05
rs5992403	2.26	0.21	8.52E-05
rs1565078	2.36	0.22	8.56E-05
rs4978629	2.38	0.23	8.62E-05
rs7576895	2.36	0.22	8.70E-05
rs8003722	2.95	0.29	8.85E-05
rs9546538	0.35	0.27	8.92E-05
rs927987	0.39	0.24	8.98E-05
rs4415146	2.33	0.22	8.98E-05
GA022530	2.46	0.24	9.01E-05
rs1029845	2.38	0.23	9.05E-05
rs2893113	6.06	0.52	9.07E-05
rs5746742	2.25	0.21	9.11E-05
rs10510535	3.99	0.39	9.12E-05
rs9902073	6.03	0.54	9.14E-05
rs458975	2.49	0.24	9.17E-05
rs9593835	0.35	0.27	9.26E-05
rs7784717	2.40	0.23	9.51E-05
rs3118752	6.34	0.56	9.60E-05

S3 Table (b). AA GWIS Associations with $P < 1x10^{-4}$ in primary model (race, sex, and principal component adjusted)

**SNP**	**Odds Ratio**	**SE**	**P**
rs13389599	22.26	1.41	7.76E-11
rs9567406	68.32	2.55	2.10E-10
rs1638665	506.92	8.19	1.04E-09
rs634138	6.10	0.64	5.46E-09
rs10979314	347.30	3.98	1.01E-08
rs3755899	1789.83	8.73	2.24E-08
rs2161719	5.76	0.42	3.81E-08
rs2989314	9.79	0.67	4.20E-08
rs9940555	7.72	0.51	4.49E-08
rs11206019	43.78	1.35	5.39E-08
rs12335314	5.91	0.70	5.39E-08
rs10046574	17.77	0.89	7.86E-08
rs9397365	21.07	0.76	8.05E-08
rs11190074	7.36	0.62	9.44E-08
rs10484876	45.75	1.27	9.94E-08
rs6570628	21.77	0.80	1.20E-07
rs7787478	7.92	0.57	1.60E-07
rs17143122	807.55	5.69	1.90E-07

rs499832	6.66	0.48	1.96E-07
rs11466653	78.65	4.55	2.28E-07
rs17019537	9.51	0.79	3.65E-07
rs2073300	12.35	0.97	3.76E-07
rs28667979	23.79	0.79	4.19E-07
rs4547347	126.36	2.20	4.65E-07
rs7022762	17.57	0.75	4.67E-07
rs7860288	17.57	0.75	4.67E-07
rs9367486	46.78	1.33	4.96E-07
rs736088	67.20	1.69	5.22E-07
rs9824246	6.20	0.52	5.78E-07
rs11077983	5.30	0.45	6.36E-07
rs16944118	6.49	0.50	6.56E-07
rs9557207	31.19	1.05	6.79E-07
rs16855732	22.59	1.05	7.09E-07
rs11942139	20.86	1.01	7.16E-07
rs9356334	87.33	2.10	7.26E-07
rs12445943	6.26	0.46	8.01E-07
rs11512640	30.22	1.18	8.65E-07
rs10428206	29.80	1.25	9.59E-07
rs11220138	31.08	1.19	9.78E-07
rs11819158	8.05	0.79	9.83E-07
rs1539189	7.35	0.68	1.01E-06
rs11039100	71.94	1.82	1.07E-06
rs9747201	5.21	0.45	1.08E-06
rs6698723	4.82	0.44	1.14E-06
rs7046999	6.15	0.62	1.15E-06
rs16837982	20.37	0.99	1.18E-06
rs10467515	35.43	1.10	1.23E-06
rs9596720	35.15	1.11	1.41E-06
rs12257301	6.19	0.45	1.45E-06
rs2056389	10.39	0.63	1.52E-06
rs10039526	13.42	0.74	1.61E-06
rs750723	120.26	4.84	1.64E-06
rs11209878	6.00	0.71	1.69E-06
rs6909762	57.27	1.68	1.72E-06
rs234284	17.97	0.81	1.73E-06
rs1846413	5.18	0.53	1.75E-06
rs4239020	5.09	0.45	1.75E-06
rs1735894	5.39	0.47	1.94E-06
rs242423	6.72	0.60	1.97E-06
rs6748245	51.10	1.36	1.99E-06
rs962073	6.08	0.59	2.02E-06
rs9600550	34.12	1.04	2.06E-06
rs17391705	50.12	1.32	2.10E-06
rs10508774	11.55	0.69	2.15E-06
rs16914101	4.90	0.44	2.24E-06
rs4798730	1764.85	3.52	2.30E-06
rs1912124	118.90	1.95	2.30E-06
rs11012350	5.11	0.52	2.31E-06
rs8121203	27.57	1.07	2.34E-06
rs1451882	14.73	0.90	2.37E-06
rs13147122	25.73	1.19	2.69E-06
rs1960306	5.15	0.53	2.82E-06
rs16919174	116.97	2.09	2.82E-06
rs2592394	5.66	0.44	2.87E-06
rs10196592	46.63	1.64	2.88E-06

rs4134872	15.74	0.80	2.98E-06
rs10508336	49.17	1.42	3.04E-06
rs2431703	23.46	1.24	3.07E-06
rs4757882	6.53	0.54	3.17E-06
rs10507495	13.68	0.86	3.20E-06
rs11771793	4.64	0.39	3.22E-06
rs10421078	26.09	1.03	3.27E-06
rs720844	4.33	0.38	3.32E-06
rs11827555	4.84	0.56	3.56E-06
rs9304301	18.99	0.94	3.91E-06
rs16965478	5.32	0.52	3.92E-06
rs12056645	5.18	0.45	4.13E-06
rs10129758	5.48	0.49	4.15E-06
rs11622263	194.87	4.64	4.27E-06
rs2281558	4.64	0.39	4.42E-06
rs12704294	6698.82	9.11	4.47E-06
rs615026	16.08	0.98	4.50E-06
rs12175863	23.28	1.27	4.51E-06
rs17307778	17.81	1.10	4.56E-06
rs11990843	5.26	0.48	4.57E-06
rs7295749	4.45	0.50	4.72E-06
rs16892422	8.76	0.72	4.96E-06
rs660323	5.58	0.44	5.09E-06
rs1450667	198.60	5.12	5.34E-06
rs256811	3.87	0.39	5.44E-06
rs11596966	78.49	1.58	5.51E-06
rs17064440	5.78	0.63	5.60E-06
rs16832236	18.32	1.06	5.67E-06
rs1027896	20.58	1.12	5.70E-06
rs1684978	3.91	0.38	5.83E-06
rs4897936	15.79	1.06	5.96E-06
rs6678735	5.76	0.69	6.08E-06
rs10115371	5.92	0.43	6.15E-06
rs17429538	37.08	1.18	6.33E-06
rs12777098	19.14	1.02	6.35E-06
rs4789729	4.31	0.40	6.37E-06
rs225872	23.61	1.18	6.39E-06
rs17725600	12.73	0.85	6.49E-06
rs1832987	47.27	1.66	6.49E-06
rs4669572	5.47	0.47	6.54E-06
rs10134584	5.23	0.49	6.80E-06
rs17130391	19.63	0.97	6.99E-06
rs1250309	10.35	0.63	7.02E-06
rs1786426	5.08	0.57	7.04E-06
rs3858306	5.11	0.43	7.19E-06
rs17037831	4.72	0.46	7.20E-06
rs6537319	12.57	0.85	7.29E-06
rs8020981	13.71	1.08	7.38E-06
rs12024301	5.05	0.57	7.56E-06
rs8073525	14.10	0.88	7.71E-06
rs9784649	14.32	0.75	7.93E-06
rs3905000	5.00	0.44	7.96E-06
rs9382099	25.29	1.24	8.36E-06
rs11971803	5.57	0.53	8.53E-06
rs12038357	33.51	1.11	8.57E-06
rs7982202	6.73	0.70	8.59E-06
rs16962242	18.29	0.88	8.62E-06

rs13335729	4.43	0.39	8.93E-06
rs6957127	10.31	0.80	8.94E-06
rs11605275	6.16	0.65	9.00E-06
rs13257525	4.07	0.37	9.15E-06
rs1593415	17.58	1.10	9.16E-06
rs1798817	4.89	0.51	9.29E-06
rs3219474	8.16	0.73	9.42E-06
rs6127146	24.00	1.12	9.58E-06
rs16981522	549.41	5.64	9.69E-06
rs4596377	36.12	1.39	9.75E-06
rs34717587	25.14	1.25	9.83E-06
rs17233103	22.75	1.26	1.00E-05
rs9365409	169.43	3.16	1.02E-05
rs12807369	5.90	0.50	1.04E-05
rs672095	10.37	0.66	1.05E-05
rs711825	5.09	0.43	1.09E-05
rs7905504	10.01	0.82	1.11E-05
rs3181225	4.23	0.47	1.11E-05
rs7232985	3.99	0.44	1.12E-05
rs11022114	13.01	0.71	1.13E-05
rs12247913	4.00	0.47	1.13E-05
rs10117316	27.46	1.36	1.15E-05
rs7830816	4.51	0.41	1.15E-05
rs16832352	21.84	1.17	1.17E-05
rs10819779	38.62	1.50	1.18E-05
rs188750	7.99	0.59	1.20E-05
rs7901466	12.05	0.69	1.21E-05
rs10131663	4.18	0.39	1.26E-05
rs17147740	7.93	0.65	1.30E-05
rs2510157	4.09	0.43	1.31E-05
rs12605132	23.13	1.05	1.33E-05
rs11915226	5.06	0.59	1.38E-05
rs7970417	8.46	0.57	1.41E-05
rs16975650	7.41	0.56	1.45E-05
rs6254	21.07	0.98	1.47E-05
rs2569254	4.17	0.47	1.48E-05
rs2391106	4.30	0.47	1.49E-05
rs9477218	49.21	1.44	1.51E-05
rs7605628	11.74	0.69	1.52E-05
rs6980740	3.80	0.43	1.54E-05
rs9481643	14.67	0.84	1.55E-05
rs12410212	42.86	1.53	1.56E-05
rs1055970	4.52	0.46	1.60E-05
rs7185036	3.99	0.38	1.61E-05
rs4919017	10.16	0.68	1.63E-05
rs9622495	5.20	0.52	1.63E-05
rs17836804	11.01	0.65	1.63E-05
rs17139138	6.57	0.49	1.69E-05
rs16910400	4.83	0.52	1.71E-05
rs7873053	4.56	0.39	1.74E-05
rs12056981	47.07	1.59	1.75E-05
rs497022	10.55	0.66	1.75E-05
rs17125553	4.68	0.46	1.76E-05
rs7093355	4.27	0.44	1.76E-05
rs13259685	4.32	0.38	1.77E-05
rs4752066	4.30	0.48	1.78E-05
rs16891597	4.67	0.46	1.78E-05

rs4714513	4.68	0.40	1.81E-05
rs10401645	10.91	0.69	1.83E-05
rs239027	3.74	0.37	1.85E-05
rs11815171	4.78	0.49	1.90E-05
rs6142859	55.02	1.53	1.91E-05
rs12137842	1749.84	5.49	1.91E-05
rs17148284	4.87	0.51	1.91E-05
rs10138933	4.94	0.49	1.93E-05
rs6685359	16.62	0.82	1.97E-05
rs12079836	4.26	0.42	1.97E-05
rs6830548	4.41	0.48	2.01E-05
rs11618570	4.06	0.40	2.03E-05
rs4368817	12.29	0.85	2.04E-05
rs17135409	3.84	0.43	2.04E-05
rs6713543	70.21	1.58	2.06E-05
rs9365855	18.16	1.09	2.07E-05
rs7316303	4.49	0.48	2.07E-05
rs17081017	13.73	0.78	2.10E-05
rs2986737	11.48	0.75	2.11E-05
rs4605886	5.51	0.55	2.13E-05
rs9819495	142.60	2.72	2.13E-05
rs4721648	0.18	0.45	2.15E-05
rs16856762	1008.42	5.37	2.21E-05
rs7207191	20.68	1.05	2.29E-05
rs9554568	15.43	0.86	2.30E-05
rs12541465	4.13	0.40	2.30E-05
rs693978	5.38	0.42	2.31E-05
rs1550558	5.37	0.45	2.31E-05
rs17047453	9.49	0.63	2.32E-05
rs6697084	9.47	0.65	2.33E-05
rs1112868	34.81	1.18	2.35E-05
rs10827150	10.59	0.76	2.40E-05
rs11646066	5.41	0.44	2.42E-05
rs35486143	43.13	4.57	2.42E-05
rs4134901	29.27	1.38	2.43E-05
rs2171475	19.18	0.90	2.43E-05
rs919953	4.57	0.46	2.52E-05
rs13283256	6.42	0.68	2.56E-05
rs9557176	15.33	0.86	2.58E-05
rs16965676	22.36	0.96	2.58E-05
rs3181718	15.49	0.87	2.59E-05
rs17469385	11.26	0.93	2.60E-05
rs9633677	11.20	0.73	2.65E-05
rs17070056	5.41	0.61	2.66E-05
rs12528404	8.05	0.69	2.67E-05
rs7980687	4.22	0.39	2.69E-05
rs901720	5.73	0.47	2.69E-05
rs11793044	4.94	0.47	2.70E-05
rs11794668	4.94	0.47	2.70E-05
rs9866608	6.43	0.52	2.71E-05
rs952932	19.19	1.04	2.72E-05
rs16868737	7.31	0.56	2.72E-05
rs17025036	4.38	0.46	2.75E-05
rs9451860	25.40	1.14	2.77E-05
rs10018408	113.41	2.12	2.78E-05
GA009992	4.51	0.43	2.79E-05
rs7563103	5.09	0.45	2.80E-05

rs12166034	7.64	0.62	2.86E-05
rs6596257	4.58	0.44	2.87E-05
rs6605557	15.23	0.99	2.92E-05
rs16904171	17.64	1.02	2.92E-05
rs2252430	11.15	0.77	2.95E-05
rs9960486	4.65	0.53	2.95E-05
rs16830097	3.64	0.40	2.98E-05
rs716478	105.95	2.00	2.99E-05
rs1342760	22.66	0.97	2.99E-05
rs10817051	11.98	0.79	3.00E-05
rs3750036	4.22	0.44	3.01E-05
rs1908750	4.73	0.66	3.01E-05
rs16965811	5.14	0.63	3.02E-05
rs17049741	4.64	0.58	3.06E-05
rs2275544	4.62	0.44	3.09E-05
rs12306432	8.04	0.57	3.09E-05
rs7195194	3.90	0.37	3.10E-05
rs4414501	3.90	0.37	3.10E-05
rs7677523	10.80	0.75	3.14E-05
rs1392372	21.80	1.18	3.18E-05
rs720840	10.25	0.68	3.22E-05
rs4943266	5.56	0.59	3.22E-05
rs11827197	11.25	0.74	3.23E-05
rs2929566	4.69	0.40	3.28E-05
rs7200988	10.67	0.67	3.29E-05
rs6913042	11.25	0.65	3.29E-05
rs10106404	11.20	0.75	3.30E-05
rs131992	4.62	0.42	3.32E-05
rs1372202	10.32	0.70	3.32E-05
rs17018642	80.56	2.05	3.33E-05
rs39986	3.43	0.38	3.39E-05
rs12640471	15.26	1.00	3.39E-05
rs7803922	4.32	0.40	3.40E-05
rs8093814	4.25	0.58	3.40E-05
rs9853287	10.97	0.76	3.40E-05
rs218237	4.05	0.37	3.43E-05
rs35779182	21.07	1.06	3.44E-05
rs2382882	4.29	0.47	3.46E-05
rs10989164	68.84	1.91	3.50E-05
rs10989163	68.84	1.91	3.50E-05
rs16919211	68.84	1.91	3.50E-05
rs7030223	4.00	0.42	3.51E-05
rs12786876	5.14	0.52	3.54E-05
rs6564716	4.58	0.48	3.58E-05
rs10190087	8.56	0.62	3.60E-05
rs749822	18.25	1.20	3.62E-05
rs7989565	80.17	1.78	3.62E-05
rs17065076	6.78	0.54	3.63E-05
rs17806552	21.14	1.17	3.64E-05
rs405031	4.50	0.40	3.64E-05
rs10026033	7.63	0.60	3.65E-05
rs1732519	5.03	0.42	3.66E-05
rs17168377	9.38	0.67	3.67E-05
rs10989165	69.03	1.92	3.70E-05
rs1810494	33.18	3.86	3.73E-05
rs7261777	4.62	0.43	3.74E-05
rs7935954	11.02	0.73	3.75E-05

rs11109007	19.34	1.07	3.75E-05
rs4837752	3.62	0.38	3.80E-05
rs17064584	5.64	0.59	3.88E-05
rs6725219	13.83	0.95	3.88E-05
rs7339053	12.18	0.72	3.92E-05
rs9396245	8.92	0.62	3.94E-05
rs9464413	8.96	0.62	3.95E-05
rs1796306	10.32	0.73	4.00E-05
rs8025995	4.91	0.61	4.00E-05
rs28496502	3.91	0.37	4.01E-05
rs7768646	11.36	0.81	4.02E-05
rs6057126	5.54	0.50	4.02E-05
rs1873380	12.31	0.93	4.05E-05
rs11602119	27.07	1.43	4.05E-05
rs1790935	4.62	0.47	4.07E-05
rs17121644	16.57	1.06	4.08E-05
rs10829791	3.95	0.41	4.10E-05
rs973954	4.02	0.43	4.19E-05
rs7655674	7.55	0.55	4.27E-05
rs6964936	4.15	0.44	4.28E-05
rs10204846	30.90	1.20	4.31E-05
rs10183184	30.90	1.20	4.31E-05
rs225475	3.84	0.44	4.31E-05
rs2977552	4.10	0.43	4.31E-05
rs10978077	4.69	0.44	4.32E-05
rs12813784	4.27	0.46	4.38E-05
rs17058330	4.99	0.52	4.44E-05
rs17156888	4.92	0.47	4.45E-05
rs28539472	3.90	0.37	4.46E-05
rs17473999	11.25	0.71	4.47E-05
rs17123327	14.33	0.93	4.47E-05
rs1488831	8.31	0.59	4.48E-05
rs7315359	4.08	0.40	4.48E-05
rs17142071	5.03	0.44	4.53E-05
rs12929663	4.45	0.54	4.57E-05
rs35987	7.23	0.63	4.57E-05
rs7185877	3.89	0.37	4.58E-05
rs11207307	12.15	0.76	4.60E-05
rs7974564	6.32	0.50	4.63E-05
rs8133734	7.18	0.61	4.63E-05
rs12915512	9.52	0.67	4.64E-05
rs17043519	13.30	0.83	4.70E-05
rs7314345	3.89	0.46	4.71E-05
rs34509814	8.96	0.67	4.72E-05
rs4436527	4.75	0.45	4.77E-05
rs7580068	10.67	0.77	4.81E-05
rs6905338	9.69	0.66	4.82E-05
rs16852893	6.60	0.65	4.85E-05
rs12769955	11.40	0.70	4.85E-05
rs11874308	3.69	0.38	4.86E-05
rs9448502	24.86	1.21	4.89E-05
rs12906810	10.71	0.67	4.89E-05
rs10835254	14.38	0.93	4.92E-05
rs16903467	17.30	1.05	4.97E-05
rs4134885	24.58	1.36	5.01E-05
rs12080418	18.74	1.09	5.02E-05
rs10860653	4.45	0.39	5.04E-05

rs16953011	21.50	1.03	5.06E-05
rs4948722	13.42	0.88	5.11E-05
rs17148598	7.45	0.56	5.12E-05
rs12598809	3.73	0.36	5.13E-05
rs13118169	8.84	0.67	5.15E-05
rs13141109	4.15	0.44	5.16E-05
rs6103601	4.13	0.43	5.18E-05
rs7164310	4.86	0.61	5.18E-05
rs10190810	17.57	0.92	5.19E-05
rs8064257	8.60	0.67	5.21E-05
rs9821348	15.06	0.99	5.25E-05
rs10001567	9.79	0.72	5.27E-05
rs10189905	3.76	0.39	5.29E-05
rs16941207	10.30	0.67	5.32E-05
rs7701189	4.62	0.54	5.33E-05
rs6789607	5.20	0.42	5.38E-05
rs7323678	4.54	0.49	5.40E-05
rs146250	3.97	0.43	5.41E-05
rs10002434	20.23	1.16	5.49E-05
rs665974	4.48	0.41	5.52E-05
rs2243133	16.67	1.14	5.53E-05
rs1054191	4.41	0.44	5.55E-05
rs6677436	4.54	0.39	5.56E-05
rs10405323	13.85	0.80	5.58E-05
rs10181159	17.00	1.05	5.61E-05
rs12574588	33.27	1.23	5.68E-05
rs10094712	10.74	0.80	5.76E-05
rs1203814	4.14	0.37	5.77E-05
rs3806708	1010.60	6.12	5.79E-05
rs13384377	3095.92	7.26	5.80E-05
rs10408321	6.55	0.69	5.83E-05
rs13275996	62.68	1.98	5.89E-05
rs17853024	13.12	0.73	5.98E-05
rs8973	4.59	0.53	6.00E-05
rs9394969	4.86	0.42	6.04E-05
rs7109515	12.33	0.93	6.08E-05
rs11890391	17.81	1.08	6.10E-05
rs6510902	3.97	0.45	6.11E-05
rs10218979	11.50	0.83	6.20E-05
rs7590083	17.65	0.93	6.21E-05
rs519800	3.98	0.49	6.22E-05
rs12419635	21.04	1.06	6.23E-05
rs725378	8.46	0.60	6.27E-05
rs17156890	4.57	0.46	6.31E-05
rs10947929	4.21	0.40	6.31E-05
rs1567318	3.65	0.38	6.34E-05
rs2014220	4.50	0.39	6.35E-05
rs10767711	13.26	0.92	6.36E-05
rs936115	14.39	0.82	6.37E-05
rs1378683	14.39	0.82	6.37E-05
rs1013870	20.16	1.23	6.43E-05
rs10497760	4.00	0.44	6.48E-05
rs9833934	11.40	0.77	6.50E-05
rs328489	9.97	0.76	6.50E-05
rs7730356	14.56	0.93	6.51E-05
rs6420969	4.21	0.53	6.52E-05
rs17129022	11.09	1.05	6.52E-05

rs7070481	4.23	0.45	6.55E-05
rs11245253	4.85	0.60	6.59E-05
rs2048917	4.25	0.40	6.61E-05
rs6430220	8.68	0.63	6.80E-05
rs16838840	6.29	0.55	6.81E-05
rs9809539	20.75	1.12	6.84E-05
rs11841493	4.19	0.40	6.84E-05
rs3741595	11.13	1.01	6.84E-05
rs907795	10.75	0.69	6.85E-05
rs2243411	8.15	0.59	6.86E-05
rs16923623	9.54	0.64	6.86E-05
rs4238255	4.04	0.42	6.87E-05
rs4910297	4.21	0.39	6.96E-05
rs4771953	11.18	0.70	6.97E-05
rs9879165	15.97	0.96	6.97E-05
rs10031329	9.46	0.63	7.07E-05
rs10178114	12.60	0.96	7.07E-05
rs12761309	12.74	0.85	7.11E-05
rs717958	12.26	0.83	7.19E-05
rs6792417	4.15	0.42	7.21E-05
rs16830291	4.15	0.42	7.21E-05
rs13421180	4.16	0.42	7.23E-05
rs571847	3.81	0.36	7.26E-05
rs222732	0.23	0.39	7.27E-05
rs7995134	4.71	0.56	7.28E-05
rs7724962	3.49	0.40	7.35E-05
rs12268694	26.82	1.42	7.37E-05
rs2339798	3.76	0.45	7.40E-05
rs6464215	4.29	0.39	7.45E-05
rs623899	57.09	1.67	7.46E-05
rs12247544	4.70	0.48	7.46E-05
rs6916164	1422138.32	9.96	7.47E-05
rs10159109	37.47	1.57	7.48E-05
rs589216	4.13	0.41	7.49E-05
rs2172011	4.27	0.43	7.51E-05
rs1017781	3.47	0.38	7.53E-05
rs7920114	3.94	0.37	7.56E-05
rs4147819	12.16	0.74	7.57E-05
rs28382829	20.09	1.23	7.58E-05
rs12047483	10.77	0.77	7.64E-05
rs9328707	3.86	0.37	7.64E-05
rs1790937	4.65	0.44	7.69E-05
rs9304276	4.42	0.51	7.72E-05
rs17109605	3.99	0.41	7.72E-05
rs12059484	4.17	0.43	7.77E-05
rs953221	307.72	7.55	7.78E-05
rs6815792	16.89	1.11	7.78E-05
rs2516698	4.00	0.43	7.79E-05
rs9582496	14.38	0.80	7.82E-05
rs7861556	4.21	0.41	7.86E-05
rs9365411	4.41	0.44	7.87E-05
rs12207132	5.48	0.69	7.87E-05
rs17039638	12.80	0.85	7.95E-05
rs35142427	4.30	0.42	7.95E-05
rs686784	4.55	0.44	7.97E-05
rs12458041	8.44	0.63	8.03E-05
rs1265045	3.23	0.35	8.05E-05

rs10021155	20.81	1.18	8.05E-05
rs28588913	9.55	0.66	8.06E-05
rs16976904	13.62	0.84	8.08E-05
rs17138263	4.46	0.49	8.09E-05
rs7902671	14.25	0.88	8.10E-05
rs3787309	4.69	0.41	8.11E-05
rs17148743	8.39	0.61	8.11E-05
rs2389672	4.20	0.44	8.19E-05
rs1055160	4.03	0.41	8.21E-05
rs7301563	11.61	0.93	8.24E-05
rs163253	7.81	0.62	8.25E-05
rs7543809	8.00	0.61	8.27E-05
rs4567801	5.98	0.51	8.29E-05
rs11466648	7.91	0.65	8.32E-05
rs11466659	7.91	0.65	8.32E-05
rs9328420	8.75	0.68	8.35E-05
rs4831658	4.66	0.48	8.36E-05
rs10173820	4.07	0.39	8.37E-05
rs327475	18.63	1.15	8.38E-05
rs6989222	6.81	0.56	8.40E-05
rs6755656	12.54	0.78	8.43E-05
rs10252263	4.34	0.40	8.43E-05
rs11912074	3.30	0.35	8.44E-05
rs12813801	4.13	0.46	8.47E-05
rs1527087	4.29	0.50	8.49E-05
rs11773898	5.38	0.54	8.55E-05
rs1574424	17.36	0.94	8.61E-05
rs283619	3.92	0.37	8.61E-05
rs10948567	21.99	1.11	8.68E-05
rs3763398	3.69	0.42	8.72E-05
rs4684731	9.30	0.76	8.80E-05
rs17396885	7.06	0.63	8.80E-05
rs10130219	3.62	0.47	8.85E-05
rs6779998	3.96	0.38	8.91E-05
rs7337274	9.37	0.69	8.91E-05
rs3824406	26.70	1.24	8.92E-05
rs3768768	14.74	0.99	8.92E-05
rs13312727	25.62	1.32	8.94E-05
rs17082453	12.76	0.96	8.95E-05
rs6901708	3.41	0.36	9.01E-05
rs11078592	7.09	0.63	9.02E-05
rs9822767	4.33	0.46	9.03E-05
rs2044457	10.08	0.71	9.04E-05
rs17050725	12.71	1.09	9.14E-05
rs738806	6.96	0.54	9.15E-05
rs1604486	4.03	0.50	9.17E-05
rs1567804	9.68	0.67	9.23E-05
rs2421139	4.71	0.40	9.23E-05
rs10405596	4.47	0.40	9.24E-05
rs12308795	59.04	1.78	9.31E-05
rs6080259	8.65	0.69	9.35E-05
rs6448490	99.78	6.44	9.40E-05
rs2000695	4.28	0.44	9.41E-05
rs2997399	338.88	9.81	9.46E-05
rs35918317	12.07	0.78	9.46E-05
rs11904214	17.45	1.14	9.48E-05
rs17769320	10.86	0.85	9.50E-05

rs17041005	12.76	1.00	9.51E-05
rs11205383	5.23	0.43	9.52E-05
rs9681462	4.20	0.43	9.55E-05
rs1333034	9.09	0.64	9.56E-05
rs6005757	6.08	0.49	9.56E-05
rs1046356	26.76	1.37	9.62E-05
rs6911846	3.67	0.37	9.68E-05
rs1119478	62.39	4.07	9.70E-05
rs1031866	60.64	4.08	9.75E-05
rs12155385	13.86	0.89	9.81E-05
rs17047540	22.10	1.20	9.84E-05
rs10213930	8.30	0.64	9.97E-05
rs17372842	12.27	0.82	9.98E-05
rs11169947	3.74	0.40	9.99E-05
rs7751276	4.25	0.45	9.99E-05
rs7874051	7347.77	7.28	1.00E-04
