# Supplementary material for: Genetic Variants in the Bone Morphogenic Protein Gene Family Modify the Association between Residential Exposure to Traffic and Peripheral Arterial Disease
Source: PLoS One. 2016 Apr 15;11(4):e0152670. doi: 10.1371/journal.pone.0152670 (PMC4833382; doi:10.1371/journal.pone.0152670)
Supplement: S4 Table — Effect Estimates for interaction, main effect, and environmental association terms for suggestive EA (a) and AA (b) GWIS variants. The odds ratio (OR), standard error (SE), and P-value (P) for the SNP-traffic exposure interaction (GxE), genetic (SNP) main effect (G), and traffic exposure (E) terms for the primary model for both the EA (a) and AA (b) suggestive (P < 1x10^-5^) variants. (PDF) [file pone.0152670.s007.pdf]

S4 Table (a). Effect Estimates for interaction, main effect, and environmental association terms for suggestive EA GWIS variants

Chrom	BP	SNP	EA GxE OR (SE)	EA GxE P	EA G OR (SE)	EA G P	EA E OR (SE)	EA E P
1	39995074	rs755249	3.45 (0.23)	2.29E-08	0.27 (0.31)	0.39	0.49 (0.22)	0.52
9	97261572	rs9409787	4.93 (0.33)	6.87E-08	0.13 (0.5)	0.19	0.62 (0.19)	0.50
1	40035686	rs17513135	3.34 (0.24)	1.33E-07	0.3 (0.31)	0.56	0.5 (0.22)	0.52
1	39731550	rs4660214	3.08 (0.24)	1.26E-06	0.28 (0.32)	0.27	0.55 (0.21)	0.52
1	39835817	rs2296172	3.08 (0.24)	1.28E-06	0.28 (0.32)	0.26	0.55 (0.21)	0.52
1	39913351	rs2296173	3.08 (0.24)	1.28E-06	0.28 (0.32)	0.26	0.55 (0.21)	0.52
1	39880319	rs3768302	3.08 (0.24)	1.28E-06	0.28 (0.32)	0.26	0.55 (0.21)	0.52
1	39815143	rs16826093	3.07 (0.24)	1.33E-06	0.28 (0.32)	0.27	0.55 (0.21)	0.52
1	39797055	rs16826069	2.99 (0.24)	1.35E-06	0.28 (0.32)	0.21	0.56 (0.21)	0.53
1	40050568	rs7539279	2.81 (0.22)	1.41E-06	0.44 (0.24)	0.91	0.41 (0.25)	0.51
1	39991588	rs3738676	2.81 (0.22)	1.51E-06	0.46 (0.24)	0.76	0.4 (0.26)	0.51
1	40044713	rs7520271	2.8 (0.22)	1.55E-06	0.44 (0.24)	0.91	0.42 (0.25)	0.52
1	39569571	rs2282231	3.07 (0.25)	1.62E-06	0.25 (0.33)	0.11	0.57 (0.2)	0.52
1	218575202	rs1317681	3.57 (0.29)	2.21E-06	0.24 (0.38)	0.26	0.62 (0.19)	0.50
9	97072647	rs2479587	7.07 (0.48)	2.26E-06	0.07 (0.79)	0.18	0.69 (0.18)	0.38
5	161997490	rs10063408	2.83 (0.23)	3.57E-06	0.47 (0.26)	0.59	0.45 (0.24)	0.51
1	40049184	rs11206378	2.64 (0.22)	5.98E-06	0.45 (0.24)	0.91	0.42 (0.26)	0.51
5	162019898	rs6879255	2.68 (0.22)	6.06E-06	0.49 (0.26)	0.51	0.45 (0.25)	0.51
6	152054374	rs12195741	2.5 (0.21)	6.32E-06	0.47 (0.25)	0.72	0.46 (0.24)	0.50
13	40297797	rs9548897	2.76 (0.23)	6.61E-06	0.58 (0.21)	0.23	0.27 (0.34)	0.48
5	161994889	rs12651722	2.66 (0.23)	7.36E-06	0.48 (0.26)	0.63	0.47 (0.24)	0.51
20	18043927	rs6045173	0.39 (0.21)	7.70E-06	2.08 (0.22)	0.92	2.36 (0.24)	0.51
1	39695155	rs10788933	2.76 (0.23)	7.72E-06	0.3 (0.28)	0.07	0.51 (0.22)	0.52
5	162023980	rs2431268	2.67 (0.23)	7.76E-06	0.49 (0.26)	0.55	0.45 (0.25)	0.51
10	88745260	rs11202287	4.31 (0.36)	8.55E-06	0.16 (0.53)	0.17	0.69 (0.18)	0.50

S4 Table (b). Effect Estimates for interaction, main effect, and environmental association terms for suggestive AA GWIS variants

Chrom	BP	SNP	AA GxE OR (SE)	AA GxE P	AA G OR (SE)	AA G P	AA E OR (SE)	AA E P
2	161732534	RS634138	6.1 (0.64)	7.67E-07	0.11 (1.11)	0.48	0.58 (0.31)	0.42
16	78805200	RS2161719	5.76 (0.42)	2.72E-06	0.09 (0.76)	0.17	0.38 (0.38)	0.46
21	21812972	RS2989314	9.79 (0.67)	2.90E-06	0.19 (0.77)	0.55	0.33 (0.45)	0.44
16	27270607	RS9940555	7.72 (0.51)	3.03E-06	0.05 (0.87)	0.11	0.4 (0.37)	0.46
6	151236180	RS9397365	21.07 (0.76)	4.46E-06	0.04 (1.15)	0.71	0.4 (0.38)	0.45
10	101121718	RS11190074	7.36 (0.62)	4.97E-06	0.04 (1.25)	0.44	0.58 (0.31)	0.43
6	144749359	RS6570628	21.77 (0.8)	5.82E-06	0.01 (1.48)	0.14	0.47 (0.35)	0.39
7	97231097	RS7787478	7.92 (0.57)	7.06E-06	0.05 (1.02)	0.26	0.46 (0.36)	0.45
10	115070140	RS499832	6.66 (0.48)	8.09E-06	0.05 (0.89)	0.01	0.46 (0.34)	0.42
