# Supplementary material for: Genetic Variants in the Bone Morphogenic Protein Gene Family Modify the Association between Residential Exposure to Traffic and Peripheral Arterial Disease
Source: PLoS One. 2016 Apr 15;11(4):e0152670. doi: 10.1371/journal.pone.0152670 (PMC4833382; doi:10.1371/journal.pone.0152670)
Supplement: S5 Table — The full list of all 25 suggestive interaction (P < 1x10^-5^) interactions from the EA GWIS. (PDF) [file pone.0152670.s008.pdf]

S5 Supplementary Table.  Consistency between EA Suggestive Results and AA results.

| Chrom | BP | SNP | OR (EA) | P (EA) | MAF (EA) | OR (AA) | P (AA) | MAF (AA) | Locus |
|---|---|---|---|---|---|---|---|---|---|
| 10 | 88745260 | rs11202287 | 4.31 | 8.55E-06 | 0.11 | 0.52 | 0.31 | 0.14 | AGAP11 |
| 1 | 39995074 | rs755249 | 3.45 | 2.29E-08 | 0.24 | | | < 0.05 | BMP8A |
| 1 | 39991588 | rs3738676 | 2.81 | 1.51E-06 | 0.36 | 0.66 | 0.35 | 0.34 | BMP8A |
| 13 | 40297797 | rs9548897 | 2.76 | 6.61E-06 | 0.49 | 1.08 | 0.85 | 0.35 | COG6 |
| 9 | 97261572 | rs9409787 | 4.93 | 6.87E-08 | 0.13 | 0.46 | 0.19 | 0.26 | DHS |
| 6 | 152054374 | rs12195741 | 2.50 | 6.32E-06 | 0.31 | 0.15 | 0.10 | 0.06 | ESR1 |
| 9 | 97072647 | rs2479587 | 7.07 | 2.26E-06 | 0.07 | 0.03 | 0.22 | 0.09 | LOC105376154 |
| 1 | 39731550 | rs4660214 | 3.08 | 1.26E-06 | 0.22 | | | < 0.05 | MACF1 |
| 1 | 39835817 | rs2296172 | 3.08 | 1.28E-06 | 0.22 | | | < 0.05 | MACF1 |
| 1 | 39880319 | rs3768302 | 3.08 | 1.28E-06 | 0.22 | | | < 0.05 | MACF1 |
| 1 | 39913351 | rs2296173 | 3.08 | 1.28E-06 | 0.22 | | | < 0.05 | MACF1 |
| 1 | 39815143 | rs16826093 | 3.07 | 1.33E-06 | 0.22 | | | < 0.05 | MACF1 |
| 1 | 39797055 | rs16826069 | 2.99 | 1.35E-06 | 0.22 | | | < 0.05 | MACF1 |
| 1 | 39569571 | rs2282231 | 3.07 | 1.62E-06 | 0.22 | 0.52 | 0.36 | 0.14 | MACF1 |
| 1 | 39695155 | rs10788933 | 2.76 | 7.72E-06 | 0.30 | 0.90 | 0.79 | 0.34 | MACF1 |
| 1 | 40035686 | rs17513135 | 3.34 | 1.33E-07 | 0.23 | | | < 0.05 | PAPBC4 |
| 1 | 218575202 | rs1317681 | 3.57 | 2.21E-06 | 0.17 | 0.71 | 0.59 | 0.15 | TGFB2 |
| 1 | 40050568 | rs7539279 | 2.81 | 1.41E-06 | 0.34 | 0.94 | 0.87 | 0.49 | |
| 1 | 40044713 | rs7520271 | 2.80 | 1.55E-06 | 0.34 | 0.99 | 0.98 | 0.50 | |
| 5 | 161997490 | rs10063408 | 2.83 | 3.57E-06 | 0.28 | 1.23 | 0.70 | 0.16 | |
| 1 | 40049184 | rs11206378 | 2.64 | 5.98E-06 | 0.37 | 1.25 | 0.59 | 0.41 | |
| 5 | 162019898 | rs6879255 | 2.68 | 6.06E-06 | 0.29 | 1.69 | 0.26 | 0.27 | |
| 5 | 161994889 | rs12651722 | 2.66 | 7.36E-06 | 0.28 | 0.38 | 0.12 | 0.24 | |
| 20 | 18043927 | rs6045173 | 0.39 | 7.70E-06 | 0.46 | 0.66 | 0.35 | 0.28 | |
| 5 | 162023980 | rs2431268 | 2.67 | 7.76E-06 | 0.29 | 1.18 | 0.68 | 0.34 | |
