# Supplementary material for: Genetic Variants in the Bone Morphogenic Protein Gene Family Modify the Association between Residential Exposure to Traffic and Peripheral Arterial Disease
Source: PLoS One. 2016 Apr 15;11(4):e0152670. doi: 10.1371/journal.pone.0152670 (PMC4833382; doi:10.1371/journal.pone.0152670)
Supplement: S6 Table — The full list of the meta-analysis results from all 34 suggestive interactions (P < 1x10^-5^) interactions from the race-stratified GWIS. (PDF) [file pone.0152670.s009.pdf]

S6 Supplementary Table. Meta-Analysis Suggestive Results

Chrom	BP	SNP	OR (EA)	P (EA)	MAF (EA)	OR (AA)	P (AA)	MAF (AA)	Meta-Analysis P	Consistent	Effect Allele
5	162019898	rs6879255	2.68	6.06E-06	0.29	1.69	0.26	0.27	7.76E-06	Yes	G
1	39995074	rs755249	3.45	2.29E-08	0.24	0.04	0.34	0.04	1.45E-05	No	G
5	161997490	rs10063408	2.83	3.57E-06	0.28	1.23	0.70	0.16	2.73E-05	Yes	G
1	40044713	rs7520271	2.80	1.55E-06	0.34	0.99	0.98	0.50	3.22E-05	Yes	C
1	40035686	rs17513135	3.34	1.33E-07	0.23	0.05	0.35	0.04	4.54E-05	No	G
1	40050568	rs7539279	2.81	1.41E-06	0.34	0.94	0.87	0.49	4.60E-05	No	G
5	162023980	rs2431268	2.67	7.76E-06	0.29	1.18	0.68	0.34	4.79E-05	Yes	G
1	39695155	rs10788933	2.76	7.72E-06	0.30	0.90	0.79	0.34	6.46E-05	Yes	G
9	97261572	rs9409787	4.93	6.87E-08	0.13	0.46	0.19	0.26	6.76E-05	No	G
13	40297797	rs9548897	2.76	6.61E-06	0.49	1.08	0.85	0.35	6.86E-05	Yes	G
1	39797055	rs16826069	2.99	1.35E-06	0.22	0.66	0.67	0.04	7.74E-05	No	G
21	21812972	rs2989314	2.21	0.08	0.07	9.79	2.90E-06	0.12	1.14E-04	Yes	C
1	218575202	rs1317681	3.57	2.21E-06	0.17	0.71	0.59	0.15	1.37E-04	No	G
1	39880319	rs3768302	3.08	1.28E-06	0.22	0.12	0.34	0.03	2.19E-04	No	C
1	39835817	rs2296172	3.08	1.28E-06	0.22	0.12	0.34	0.03	2.19E-04	No	G
1	39913351	rs2296173	3.08	1.28E-06	0.22	0.12	0.33	0.04	2.32E-04	No	G
1	39815143	rs16826093	3.07	1.33E-06	0.22	0.12	0.33	0.04	2.39E-04	No	t
1	39991588	rs3738676	2.81	1.51E-06	0.36	0.66	0.35	0.34	2.39E-04	No	C
1	39569571	rs2282231	3.07	1.62E-06	0.22	0.52	0.36	0.14	2.39E-04	No	G
1	39731550	rs4660214	3.08	1.26E-06	0.22	0.12	0.31	0.04	2.44E-04	No	G
1	40049184	rs11206378	2.64	5.98E-06	0.37	1.25	0.59	0.41	2.84E-04	No	G
9	97072647	rs2479587	7.07	2.26E-06	0.07	0.03	0.22	0.09	5.52E-04	No	C
20	18043927	rs6045173	0.39	7.70E-06	0.46	0.66	0.35	0.28	7.06E-04	No	G
10	88745260	rs11202287	4.31	8.55E-06	0.11	0.52	0.31	0.14	8.77E-04	No	G
6	144749359	rs6570628	1.62	0.32	0.05	21.77	5.82E-06	0.12	0.002	Yes	G
5	161994889	rs12651722	2.66	7.36E-06	0.28	0.38	0.12	0.24	0.002	No	G
6	152054374	rs12195741	2.50	6.32E-06	0.31	0.15	0.10	0.06	0.002	No	G
16	27270607	rs9940555	1.59	0.58	0.02	7.72	3.03E-06	0.17	0.004	Yes	G
4	39529700	rs3755899	1.18	0.64	0.06	1789.83	1.92E-06	0.08	0.005	Yes	G
2	161732534	rs634138	0.44	1.00	0.00	6.10	7.67E-07	0.08	0.01	No	G
16	78805200	rs2161719	0.86	0.60	0.14	5.76	2.72E-06	0.18	0.05	No	G
13	44877318	rs9567406	0.66	0.27	0.13	68.32	9.83E-08	0.11	0.08	No	G
10	101121718	rs11190074	0.69	0.36	0.09	7.36	4.97E-06	0.09	0.13	No	G
10	115070140	rs499832	0.70	0.36	0.09	6.66	8.09E-06	0.20	0.14	No	C
7	97231097	rs7787478	0.47	0.09	0.10	7.92	7.06E-06	0.13	0.43	No	G
6	151236180	rs9397365	0.37	0.004	0.15	21.07	4.46E-06	0.09	0.88	No	G
