# Supplementary material for: Genetic Variants in the Bone Morphogenic Protein Gene Family Modify the Association between Residential Exposure to Traffic and Peripheral Arterial Disease
Source: PLoS One. 2016 Apr 15;11(4):e0152670. doi: 10.1371/journal.pone.0152670 (PMC4833382; doi:10.1371/journal.pone.0152670)
Supplement: S7 Table — (PDF) [file pone.0152670.s010.pdf]

S7 Table. Interaction results for all loci tested in the bone morphogenic protein family of genes (BMP1, BMP2, BMP4, BMP7, BMP8A, BMP9, BMP10, and BMPER).

BMP1										
CHROM	BP	PROBE	OR (EA)	SE (EA)	P (EA)	MAF (EA)	OR (AA)	SE (AA)	P (AA)	MAF (AA)
8	22024523	rs10096505	1.06	0.21	7.84E-01	0.48	0.84	0.43	7.24E-01	0.36
8	22025644	rs7812993	1.16	0.27	5.68E-01	0.22	0.71	0.44	4.85E-01	0.29
8	22029129	rs10095410	0.00	6364.57	9.75E-02	< 0.01	1.04	0.55	9.44E-01	0.09
8	22033435	rs11777932	1.08	0.45	8.59E-01	0.08	0.29	1.70	4.81E-01	0.03
8	22038952	rs7838961	1.16	0.22	5.00E-01	0.49	1.04	0.39	9.27E-01	0.36
8	22042151	rs7819541	0.61	0.63	4.34E-01	0.07	1.33	0.38	4.93E-01	0.46
8	22042197	rs4507761	1.12	0.39	7.68E-01	0.08	1.24	1746.86	9.99E-01	0.01
8	22046829	rs11994254				<0.01	1.48	0.65	5.85E-01	0.04
8	22048790	rs12114940	1.01	0.22	9.70E-01	0.43	0.94	0.46	9.11E-01	0.26
8	22049780	rs3924231	0.88	0.36	7.25E-01	0.17	0.89	0.39	7.94E-01	0.47
8	22049892	rs3924229	1.11	0.42	8.10E-01	0.11	0.61	0.46	3.32E-01	0.23
8	22051849	rs6997623	0.52	2333.79	9.99E-01	0.00	0.60	0.62	4.52E-01	0.16
8	22053123	rs4075478	1.01	0.23	9.74E-01	0.38	1.02	0.34	9.59E-01	0.41
8	22054197	rs12675993	1.16	0.41	7.22E-01	0.10	1.21	0.61	7.78E-01	0.11

8	22055348	rs3857979	1.07	0.22	7.69E-01	0.48	1.07	0.38	8.81E-01	0.33
8	22057449	rs7827382	0.58	0.30	7.27E-02	0.22	1.36	0.47	5.55E-01	0.22
8	22058122	rs11775186	0.78	0.44	5.69E-01	0.09	1.03	0.67	9.63E-01	0.09
8	22058904	rs11784926	0.34	1.00	2.51E-01	0.04	0.02	2.89	2.60E-01	0.02
8	22059363	rs11996036	0.80	0.49	6.45E-01	0.06	0.93	0.66	9.19E-01	0.09
8	22059413	rs28710878	0.00	4794.06	7.24E-02	< 0.01	1.64	0.49	3.60E-01	0.10
8	22062432	rs10107545	0.93	1066.24	9.98E-01	< 0.01	1.07	0.80	9.43E-01	0.09
8	22063438	rs6983732	0.50	0.34	3.87E-02	0.13	1.25	0.37	5.80E-01	0.27
8	22067769	rs4242430	0.79	0.27	3.83E-01	0.31	0.84	0.72	8.30E-01	0.09
8	22067774	rs4076873	0.92	0.25	7.25E-01	0.24	1.08	0.39	8.63E-01	0.47
8	22069018	rs11778448	0.94	0.25	8.00E-01	0.24	1.74	0.39	1.94E-01	0.34
BMP2										
CHROM	BP	PROBE	OR (EA)	SE (EA)	P (EA)	MAF (EA)	OR (AA)	SE (AA)	P (AA)	MAF (AA)
20	6751034	rs1049007	0.56	0.23	1.10E-02	0.38	0.39	0.78	2.54E-01	0.12
20	6751316	rs7270163	1.87	0.30	3.44E-02	0.11	3.56	0.39	2.51E-03	0.31
20	6754246	rs235764	1.71	0.23	1.98E-02	0.27	3.58	0.42	5.46E-03	0.41
20	6755598	rs235767	1.68	0.22	1.84E-02	0.39	3.66	0.49	1.59E-02	0.48
20	6756148	rs1005464	1.10	0.25	7.21E-01	0.22	0.26	1.05	2.24E-01	0.10
20	6759115	rs235768	0.54	0.23	7.28E-03	0.39	0.13	1.36	1.28E-01	0.06

20	6759980	rs15705	1.38	0.26	2.10E-01	0.22	1.28	0.49	6.51E-01	0.23
20	6760201	rs3178250	1.40	0.27	2.22E-01	0.19	1.53	0.49	4.29E-01	0.21
20	6760431	rs170986	1.37	0.27	2.35E-01	0.17	2.49	0.39	2.97E-02	0.31
BMP4										
CHROM	BP	PROBE	OR (EA)	SE (EA)	P (EA)	MAF (EA)	OR (AA)	SE (AA)	P (AA)	MAF (AA)
14	54417522	rs17563	1.36	0.23	1.72E-01	0.44	0.66	0.48	4.30E-01	0.24
14	54418411	rs2071047	0.66	0.23	7.58E-02	0.41	0.97	0.43	9.56E-01	0.39
14	54420653	rs10140018	1.01	1.11	9.90E-01	0.01	0.38	2.62	7.42E-01	0.03
BMP7										
CHROM	BP	PROBE	OR (EA)	SE (EA)	P (EA)	MAF (EA)	OR (AA)	SE (AA)	P (AA)	MAF (AA)
20	55743905	rs12438	1.26	0.22	2.98E-01	0.49	2.10	0.37	6.22E-02	0.25
20	55744499	rs6014947	1.21	0.22	3.82E-01	0.49	1.87	0.39	1.27E-01	0.21
20	55749781	rs6025422	1.20	0.22	4.08E-01	0.50	1.77	0.36	1.46E-01	0.39
20	55753082	rs6025427	0.14	2.55	3.88E-01	< 0.01	1.12	0.59	8.64E-01	0.14
20	55754110	rs3787380	1.03	0.22	8.82E-01	0.39	0.63	0.43	3.23E-01	0.22
20	55754239	rs13037653	0.50	0.49	1.48E-01	0.07	0.13	2.41	3.79E-01	0.01
20	55755170	rs3787382	1.05	0.28	8.53E-01	0.19	0.87	0.36	7.29E-01	0.49
20	55756841	rs6099488	0.82	5355.63	9.99E-01	< 0.01	3.22	1.18	3.53E-01	0.03
20	55756881	rs6025428	1.82	1576.23	9.98E-01	< 0.01	0.68	0.51	4.85E-01	0.13

20	55758655	rs6070015	0.99	0.50	9.80E-01	0.05	1.25	0.34	5.54E-01	0.48
20	55760567	rs6064508	0.77	0.24	2.84E-01	0.37	0.52	0.55	2.72E-01	0.16
20	55761285	rs1318379	1.12	0.48	8.13E-01	0.06	1.03	0.36	9.36E-01	0.36
20	55762947	rs6123674	1.24	0.23	3.53E-01	0.42	1.19	0.41	6.98E-01	0.21
20	55763739	rs6099492	0.50	0.84	4.04E-01	0.03	1.49	0.42	3.97E-01	0.14
20	55764104	rs6064509	1.24	0.73	7.71E-01	0.02	5.78	2433.94	9.99E-01	0.00
20	55772683	rs230191	1.37	0.22	1.57E-01	0.49	0.57	0.45	2.59E-01	0.24
20	55776447	rs230198	0.67	0.22	7.28E-02	0.46	2.02	0.38	9.51E-02	0.38
20	55785618	rs6123677	1.39	0.22	1.41E-01	0.49	0.76	0.48	6.00E-01	0.14
20	55786811	rs6025446	0.99	0.22	9.79E-01	0.42	1.04	0.35	9.17E-01	0.46
20	55787622	rs12481628	0.63	0.22	3.66E-02	0.44	1.12	0.37	7.90E-01	0.33
20	55788009	rs10470036	0.55	2.44	8.04E-01	< 0.01	3.20	0.47	7.46E-03	0.08
20	55789914	rs3764677	0.58	0.23	1.91E-02	0.42	1.49	0.36	3.10E-01	0.35
20	55790147	rs6025447	0.56	0.23	1.26E-02	0.40	0.53	0.76	4.38E-01	0.16
20	55791348	rs6123679	2.50	0.72	1.75E-01	0.03	0.49	1.75	7.02E-01	0.01
20	55792300	rs1002432	1.07	0.61	9.09E-01	0.03	1.21	0.36	6.30E-01	0.37
20	55794749	rs46995	0.63	0.23	3.97E-02	0.43	0.38	0.71	1.99E-01	0.20
20	55797057	rs6127972	0.58	0.23	1.62E-02	0.41	0.52	0.70	3.90E-01	0.19
20	55800039	rs172983	1.42	0.31	2.47E-01	0.11	0.59	0.56	3.90E-01	0.17

20	55802736	rs162311	1.38	0.29	2.70E-01	0.13	0.56	0.39	1.71E-01	0.42
20	55802858	rs162312	1.49	0.27	1.38E-01	0.17	0.67	0.43	3.99E-01	0.34
20	55803687	rs17404303	1.28	0.22	2.66E-01	0.41	1.66	0.48	3.34E-01	0.12
20	55805127	rs2180782	1.43	0.26	1.68E-01	0.17	0.53	0.40	1.59E-01	0.43
20	55806280	rs6127973	0.77	0.33	4.39E-01	0.15	0.00	5.31	1.27E-01	0.03
20	55806340	rs186659	0.59	0.23	2.08E-02	0.40	0.32	0.95	2.63E-01	0.11
20	55807110	rs6014959	1.75	0.32	7.61E-02	0.13	1.24	0.38	6.16E-01	0.45
20	55809072	rs1028644	1.71	0.24	2.34E-02	0.25	1.00	0.45	9.93E-01	0.31
20	55811812	rs6025456	2.41	1.81	6.22E-01	0.01	1.58	0.35	2.42E-01	0.25
20	55812058	rs7273197	1.01	0.23	9.81E-01	0.31	1.79	0.49	2.84E-01	0.13
20	55816734	rs4361186	3.91	1.71	4.10E-01	0.01	1.68	0.36	1.97E-01	0.33
20	55824475	rs6127983	1.23	0.22	3.54E-01	0.35	0.88	0.37	7.61E-01	0.42
20	55824533	rs182784	0.64	0.22	3.98E-02	0.48	0.28	0.58	4.04E-02	0.28
20	55824760	rs6127985	1.26	0.28	4.10E-01	0.16	0.70	0.46	4.82E-01	0.24
20	55830151	rs6025468	1.04	0.27	8.85E-01	0.19	2.31	0.35	2.93E-02	0.29
20	55834657	rs7269453				< 0.01	1.14	1.00	9.07E-01	0.07
BMP9										
CHROM	BP	PROBE	OR (EA)	SE (EA)	P (EA)	MAF (EA)	OR (AA)	SE (AA)	P (AA)	MAF (AA)
10	48413273	rs3740297	0.89	794.32	9.98E-01	0.01	0.68	1.59	8.25E-01	0.01

BMP10										
CHROM	BP	PROBE	OR (EA)	SE (EA)	P (EA)	MAF (EA)	OR (AA)	SE (AA)	P (AA)	MAF (AA)
2	69092621	rs10173546				< 0.01	1.34	0.60	6.48E-01	0.12
2	69093312	rs2231344	2.37	0.39	2.26E-02	0.07	0.60	0.48	3.28E-01	0.25
2	69095152	rs2312078	1.11	0.22	6.25E-01	0.39	0.73	0.40	4.75E-01	0.45
2	69097862	rs3817368	1.12	0.22	6.08E-01	0.39	0.32	0.57	6.96E-02	0.27
2	69097953	rs6760348	1.65E+49	3536.57	8.80E-02	< 0.01	0.61	0.82	5.77E-01	0.09
BMP8A										
CHROM	BP	PROBE	OR (EA)	SE (EA)	P (EA)	MAF (EA)	OR (AA)	SE (AA)	P (AA)	MAF (AA)
1	39963873	rs2004330	0.46	0.23	5.74E-04	0.44	1.39	0.45	5.11E-01	0.25
1	39965761	rs698141	0.98	0.43	9.63E-01	0.06	0.80	0.86	8.16E-01	0.08
1	39966718	rs6656934	0.78	0.36	4.95E-01	0.11	1.04	0.35	9.11E-01	0.47
1	39972874	rs11206218	0.43	0.97	3.73E-01	0.03	1.68	0.49	3.35E-01	0.18
1	39973646	rs782869	0.91	1.14	9.37E-01	< 0.01	0.40	0.66	1.91E-01	0.14
1	39975894	rs1180327	0.11	1518.03	9.98E-01	< 0.01	0.02	4.33	4.10E-01	0.04
1	39989012	rs11584399	0.90	0.35	7.55E-01	0.09	0.91	0.74	9.13E-01	0.05
1	39989926	rs710913	2.42	0.21	3.04E-05	0.36	2.11	0.38	7.33E-02	0.31
1	39991588	rs3738676	2.81	0.22	1.51E-06	0.36	0.66	0.39	3.47E-01	0.34
1	39992537	rs1180340	0.57	718.36	9.98E-01	< 0.01	1.91	0.44	1.58E-01	0.12

1	39992808	rs1180341	0.44	0.22	1.24E-04	0.46	0.82	0.38	6.37E-01	0.32
1	39993432	rs1180343	0.43	0.23	1.74E-04	0.45	1.50	0.46	4.29E-01	0.24
1	39995074	rs755249	3.45	0.23	2.29E-08	0.24	0.04	3.19	3.39E-01	0.04
BMPER										
CHROM	BP	PROBE	OR (EA)	SE (EA)	P (EA)	MAF (EA)	OR (AA)	SE (AA)	P (AA)	MAF (AA)
7	33949959	rs7801918	0.88	0.28	6.56E-01	0.23	0.97	0.45	9.49E-01	0.26
7	33957895	rs4720146	0.21	3.52	6.13E-01	< 0.01	0.13	1.98	3.15E-01	0.01
7	33959239	rs1427483	0.72	0.23	1.39E-01	0.37	1.27	0.43	6.24E-01	0.28
7	33967085	rs13221600	0.60	0.25	3.63E-02	0.25	0.53	0.73	4.24E-01	0.12
7	33971904	rs2016770	2.19	0.24	1.12E-03	0.34	1.57	0.41	3.20E-01	0.42
7	33972999	rs747664	1.04	685.08	9.99E-01	0.00	0.13	3.34	4.99E-01	0.02
7	33973653	rs10253162	2.38	0.25	4.29E-04	0.34	1.26	0.39	5.95E-01	0.45
7	33977757	rs10247996	0.70	0.23	1.18E-01	0.46	0.81	0.45	6.71E-01	0.31
7	33979694	rs10486624	2.96	1345.73	9.98E-01	< 0.01	1.72	0.56	3.79E-01	0.09
7	33984313	rs1427481	0.85	0.23	4.87E-01	0.45	0.88	0.39	7.59E-01	0.49
7	33991109	rs13233238	0.57	0.24	2.06E-02	0.28	1.36	0.41	4.96E-01	0.46
7	33991875	rs10270579	0.57	0.24	1.79E-02	0.29	1.53	0.38	3.15E-01	0.47
7	33992983	rs1365889	0.77	0.40	5.16E-01	0.12	0.58	1.04	6.29E-01	0.03
7	33996948	rs12155213	0.63	0.24	4.78E-02	0.31	1.44	0.37	3.75E-01	0.47

7	33998587	rs4723343	1.32	0.22	2.16E-01	0.42	2.76	0.43	3.25E-02	0.37
7	34000224	rs9639688	1.41	0.23	1.37E-01	0.23	0.25	0.70	5.17E-02	0.17
7	34009946	rs10265207	1.05	0.22	8.28E-01	0.47	1.96	0.36	8.42E-02	0.38
7	34012829	rs1365888	0.71	0.39	3.80E-01	0.12	0.60	0.88	6.02E-01	0.08
7	34013178	rs13243958	1.20	0.22	3.99E-01	0.35	0.50	0.41	1.25E-01	0.42
7	34017577	rs10276432	1.07	0.22	7.57E-01	0.35	0.67	0.41	3.75E-01	0.42
7	34021755	rs12701341	0.91	0.22	6.63E-01	0.40	0.66	0.40	3.50E-01	0.46
7	34022301	rs10486625	1.12	0.22	6.06E-01	0.37	0.54	0.37	1.28E-01	0.49
7	34024288	rs6951556				< 0.01	0.06	2.78	3.33E-01	0.01
7	34035784	rs6948650	1.17	0.39	6.87E-01	0.07	0.45	0.78	3.40E-01	0.09
7	34036618	rs9986855	0.81	0.30	4.76E-01	0.15	0.45	1.05	4.62E-01	0.03
7	34038414	rs17169617	0.90	0.29	7.04E-01	0.17	0.40	0.56	1.39E-01	0.22
7	34043348	rs10951405	0.59	0.30	7.34E-02	0.18	0.91	0.56	8.86E-01	0.14
7	34046002	rs974937	1.37	0.25	1.96E-01	0.22	0.63	0.42	3.21E-01	0.40
7	34074661	rs12670628	0.94	0.23	7.83E-01	0.31	0.61	0.37	2.30E-01	0.47
7	34087546	rs17169630	0.95	0.39	8.91E-01	0.08	0.78	0.43	6.00E-01	0.34
7	34088690	rs17823423	0.86	0.26	5.51E-01	0.23	0.45	1.45	6.11E-01	0.04
7	34089823	rs7806522	0.93	0.23	7.73E-01	0.30	0.57	0.39	1.88E-01	0.47
7	34090734	rs13244436	0.96	0.25	8.66E-01	0.23	0.54	0.69	4.23E-01	0.09

7	34094687	rs17169636	1.09	0.40	8.19E-01	0.07	1.14	0.51	8.17E-01	0.15
7	34103553	rs1420328	0.75	0.40	4.71E-01	0.10	2.08	0.43	1.20E-01	0.22
7	34107393	rs1362457	0.98	0.25	9.32E-01	0.22	0.58	0.63	4.31E-01	0.13
7	34111660	rs961652	0.85	0.22	4.65E-01	0.48	1.18	0.41	7.19E-01	0.29
7	34117136	rs759417	1.23	0.22	3.46E-01	0.29	1.51	0.36	3.01E-01	0.48
7	34121829	rs2160308	1.14	0.23	5.65E-01	0.29	0.81	0.47	6.86E-01	0.17
7	34125622	rs10249320	1.21	720.50	9.98E-01	0.01			< 0.01	
7	34126782	rs7790026	1.23	0.22	3.45E-01	0.30	1.36	0.35	4.31E-01	0.49
7	34128156	rs11973757	0.24	2052.01	9.99E-01	< 0.01	7.18	0.73	4.26E-03	0.06
7	34132131	rs1420331	1.09	0.24	7.32E-01	0.27	0.59	0.45	2.82E-01	0.28
7	34132727	rs1420332	1.08	0.24	7.39E-01	0.27	1.38	0.39	4.51E-01	0.50
7	34133050	rs973766	1.76	0.23	1.32E-02	0.31	0.17	1.48	2.71E-01	0.06
7	34133861	rs10261012	1.09	0.24	7.29E-01	0.27	0.64	0.43	3.50E-01	0.32
7	34134582	rs1420333	1.09	0.24	7.13E-01	0.27	1.11	0.39	8.11E-01	0.39
7	34135625	rs12701343	1.09	0.26	7.26E-01	0.23	0.16	1.29	1.69E-01	0.08
7	34137529	rs6948894	1.12	0.24	6.50E-01	0.27	0.62	0.44	3.31E-01	0.32
7	34137970	rs12701344	1.25	729.85	9.98E-01	< 0.01	2.67	0.86	2.89E-01	0.07
7	34138717	rs1946146	1.03	0.25	8.93E-01	0.23	0.54	0.75	4.49E-01	0.11
7	34140513	rs1420335	0.96	0.24	8.51E-01	0.31	1.27	0.40	5.95E-01	0.39

7	34140927	rs6462526	0.96	0.24	8.72E-01	0.31	0.99	0.37	9.89E-01	0.47
7	34143378	rs1420339	0.38	669.41	9.98E-01	0.00	1.12	0.81	9.02E-01	0.06
7	34147159	rs10271544	1.06	0.21	7.81E-01	0.40	0.79	0.40	5.89E-01	0.40
7	34151057	rs757640	1.27	0.25	3.37E-01	0.19	1.14	0.39	7.62E-01	0.34
7	34153768	rs918038	1.08	0.21	7.03E-01	0.39	0.79	0.38	5.69E-01	0.44
7	34156746	rs2080372	1.20	0.25	4.78E-01	0.19	0.81	0.50	7.00E-01	0.19
7	34157300	rs17169653	2.64	1676.17	9.98E-01	< 0.01	0.63	0.99	6.71E-01	0.07
7	34158292	rs10236500	0.91	2466.61	9.98E-01	< 0.01	3.89	0.75	6.04E-02	0.07
7	34159138	rs1345347	1.19	0.25	4.85E-01	0.19	1.29	0.41	5.78E-01	0.27
7	34164659	rs1014580	1.22	0.25	4.43E-01	0.19	1.34	0.41	5.23E-01	0.29
7	34164677	rs1014581	1.08	0.21	7.12E-01	0.39	0.81	0.38	6.05E-01	0.45
7	34167361	rs10486631	0.89	0.28	6.81E-01	0.19	0.26	0.89	1.45E-01	0.10
7	34168028	rs6462533	1.05	0.21	8.28E-01	0.40	0.82	0.40	6.50E-01	0.43
7	34169964	rs1861362	1.22	0.25	4.30E-01	0.19	0.47	0.57	2.32E-01	0.21
7	34173852	rs10951406	1.15	0.25	5.94E-01	0.20	1.04	0.38	9.24E-01	0.46
7	34174173	rs6961966	0.95	0.28	8.45E-01	0.20	0.43	0.65	2.35E-01	0.16
7	34178283	rs12673452	0.03	5.32	3.96E-01	0.01	1.03	0.51	9.63E-01	0.12
7	34178645	rs10486632	1.16	0.25	5.70E-01	0.20	0.62	0.41	2.96E-01	0.32
7	34191345	rs1014578	1.03	0.21	8.79E-01	0.40	0.77	0.37	5.15E-01	0.42

7	34192011	rs918037	1.13	0.21	5.75E-01	0.40	0.60	0.38	2.23E-01	0.41
7	34192762	rs6968741	1.34	0.34	3.87E-01	0.06	1.22	0.36	6.24E-01	0.17
7	34193589	rs11972404	1.85	0.61	3.02E-01	0.03	0.90	0.43	8.23E-01	0.23
7	34193952	rs17169667	1.09	0.33	7.93E-01	0.11	1.64	0.40	2.63E-01	0.49
7	34194102	rs2058681	1.24	0.22	3.30E-01	0.32	0.77	0.92	7.94E-01	0.06
