# Supplementary material for: Genetic Variants in the Bone Morphogenic Protein Gene Family Modify the Association between Residential Exposure to Traffic and Peripheral Arterial Disease
Source: PLoS One. 2016 Apr 15;11(4):e0152670. doi: 10.1371/journal.pone.0152670 (PMC4833382; doi:10.1371/journal.pone.0152670)
Supplement: S8 Table — This table gives the eQTL associations for all genes associated with variants in BMP8A with an interaction P < 0.05 in the EA GWIS. Data on the eQTLs was taken from the GTEx Release V6[37]. (PDF) [file pone.0152670.s011.pdf]

S8 Table. Cis-eQTL associations for all genes associated with variants in BMP8A (EA GWIS P < 0.05). This table gives the eQTL associations for all genes associated with variants in BMP8A with an interaction P < 0.05 in the EA GWIS. Data on the eQTLs was taken from the GTEx Release V6[33].

BMP8A eQTLs		rs3738676	rs2004330	rs1180343	rs1180341	rs755249	rs710913
	Adipose - Subcutaneous	$3.7x10^{-6}$ (0.37)					$1.7x10^{-5}$ (-0.33)
	Artery - Tibial				$5.3x10^{-6}$ (-0.36)		
	Brain - Cortex				$3.7x10^{-6}$ (-0.44)		
	Esophagus - Muscularis		$8.6x10^{-6}$ (-0.37)	$1x10^{-5}$ (-0.38)	$2.3x10^{-6}$ (-0.4)	$1.5x10^{-5}$ (0.44)	$5.5x10^{-6}$ (-0.38)
	Heart - Atrial Appendage		$1.3x10^{-9}$ (-0.52)	$1.2x10^{-9}$ (-0.54)	$8.8x10^{-10}$ (-0.54)		
	Heart - Left Ventricle				$6.6x10^{-6}$ (-0.42)		
	Nerve - Tibial	$1.2x10^{-5}$ (0.2)	$5.5x10^{-7}$ (-0.25)	$1.3x10^{-6}$ (-0.24)	$2.6x10^{-6}$ (-0.23)	$3.6x10^{-6}$ (0.25)	
	Testis	$2.1x10^{-12}$ (0.54)	$6.9x10^{-11}$ (-0.5)	$2.2x10^{-10}$ (-0.48)	$2.3x10^{-16}$ (-0.61)	$2x10^{-7}$ (0.5)	$9.5x10^{-12}$ (-0.52)
OXCT2 eQTLs		rs3738676	rs2004330	rs1180343	rs1180341	rs755249	rs710913
	Thyroid		$1.6x10^{-6}$ (0.3)	$1.2x10^{-6}$ (0.31)	$7.3x10^{-6}$ (0.29)		
RP11-69E11.4 eQTLs		rs3738676	rs2004330	rs1180343	rs1180341	rs755249	rs710913
	Adipose - Subcutaneous	$1.1x10^{-9}$ (0.41)	$6.7x10^{-6}$ (-0.32)	$1.5x10^{-6}$ (-0.34)	$3.1x10^{-11}$ (-0.44)	$8.4x10^{-9}$ (0.46)	$1.2x10^{-7}$ (-0.34)
	Adipose - Visceral (Omentum)	$3.2x10^{-8}$ (0.46)			$1.4x10^{-7}$ (-0.42)		$1.3x10^{-6}$ (-0.4)
	Artery - Aorta	$1.6x10^{-8}$ (0.41)		$9.5x10^{-6}$ (-0.32)	$2x10^{-6}$ (-0.33)	$9.9x10^{-8}$ (0.41)	$3.4x10^{-7}$ (-0.35)

	Artery - Tibial	6.5×10^{-16} (0.46)	1.8×10^{-7} (-0.29)	1.1×10^{-7} (-0.3)	4.8×10^{-13} (-0.42)	1.1×10^{-10} (0.44)	1.5×10^{-11} (-0.39)
	Brain - Cerebellar Hemisphere	7.4×10^{-6} (0.4)					
	Brain - Frontal Cortex (BA9)	3.6×10^{-6} (0.41)					
	Breast - Mammary Tissue	3.1×10^{-8} (0.44)			3.9×10^{-8} (-0.42)		7.6×10^{-7} (-0.38)
	Cells - Transformed fibroblasts	4.3×10^{-13} (0.44)	9.2×10^{-9} (-0.34)	1.1×10^{-8} (-0.34)	7.6×10^{-13} (-0.41)	2×10^{-8} (0.4)	8.6×10^{-10} (-0.36)
	Colon - Sigmoid				2.2×10^{-7} (-0.37)		
	Colon - Transverse	1.7×10^{-8} (0.44)			2.4×10^{-6} (-0.36)	1.3×10^{-8} (0.56)	9.4×10^{-7} (-0.39)
	Esophagus - Gastroesophageal Junction	2.1×10^{-8} (0.4)	1.1×10^{-6} (-0.33)	6.2×10^{-8} (-0.36)	2.2×10^{-12} (-0.45)		5×10^{-9} (-0.39)
	Esophagus - Mucosa	3.3×10^{-9} (0.31)			5.5×10^{-7} (-0.27)	1.1×10^{-7} (0.32)	6.1×10^{-9} (-0.29)
	Esophagus - Muscularis	9.7×10^{-13} (0.41)	5.5×10^{-11} (-0.37)	1.4×10^{-11} (-0.39)	4.6×10^{-16} (-0.45)	4.3×10^{-10} (0.43)	1.7×10^{-12} (-0.4)
	Heart - Atrial Appendage		9.4×10^{-8} (-0.36)	3.5×10^{-8} (-0.39)	3.2×10^{-7} (-0.36)		2.5×10^{-6} (-0.35)
	Heart - Left Ventricle	1.1×10^{-7} (0.31)			6.8×10^{-9} (-0.34)		1.5×10^{-8} (-0.33)
	Lung	1.9×10^{-9} (0.37)			1.2×10^{-6} (-0.31)	3.3×10^{-8} (0.4)	2.9×10^{-9} (-0.36)
	Nerve - Tibial	1.7×10^{-11} (0.41)	1×10^{-7} (-0.35)	1.9×10^{-8} (-0.37)	3.6×10^{-11} (-0.41)	1.6×10^{-11} (0.47)	3.7×10^{-10} (-0.37)
	Pancreas	6.2×10^{-6} (0.32)					
	Skin - Not Sun Exposed (Suprapubic)	1.2×10^{-6} (0.34)			3.9×10^{-8} (-0.37)		
	Skin - Sun Exposed (Lower leg)	1.1×10^{-13} (0.43)			2.1×10^{-10} (-0.36)	1.1×10^{-8} (0.39)	1×10^{-9} (-0.34)

	Spleen	$3.6x10^{-7}$ (0.58)			$5.7x10^{-6}$ (-0.48)		$7.4x10^{-8}$ (-0.57)
	Stomach	$4.7x10^{-11}$ (0.51)			$3.9x10^{-7}$ (-0.41)	$1.5x10^{-8}$ (0.55)	$2.3x10^{-7}$ (-0.42)
	Thyroid	$6.8x10^{-8}$ (0.28)				$2.2x10^{-6}$ (0.3)	$8.5x10^{-8}$ (-0.28)
	Whole Blood	$1.1x10^{-8}$ (0.27)				$8.6x10^{-8}$ (0.29)	$1x10^{-6}$ (-0.23)
PABPC4 eQTLs		rs3738676	rs2004330	rs1180343	rs1180341	rs755249	rs710913
	Adipose - Subcutaneous					$3x10^{-8}$ (-0.28)	
	Adipose - Visceral (Omentum)					$5.5x10^{-6}$ (-0.25)	
	Artery - Tibial	$4.5x10^{-6}$ (-0.2)				$6.4x10^{-12}$ (-0.35)	
	Breast - Mammary Tissue					$6x10^{-7}$ (-0.32)	
	Cells - Transformed fibroblasts					$5.8x10^{-9}$ (-0.23)	
	Esophagus - Mucosa	$5.5x10^{-6}$ (-0.24)				$2.1x10^{-7}$ (-0.3)	
	Lung	$2.9x10^{-6}$ (-0.2)				$8.5x10^{-6}$ (-0.23)	$5x10^{-6}$ (0.19)
	Nerve - Tibial	$6.4x10^{-6}$ (-0.18)			$8.5x10^{-7}$ (0.2)	$8.2x10^{-10}$ (-0.27)	$2.5x10^{-9}$ (0.22)
	Skin - Not Sun Exposed (Suprapubic)					$1.7x10^{-7}$ (-0.29)	
	Skin - Sun Exposed (Lower leg)					$1.3x10^{-9}$ (-0.27)	$8.4x10^{-6}$ (0.17)
	Thyroid	$1.0x10^{-5}$ (-0.23)					
OXCT2P1 eQTLs		rs3738676	rs2004330	rs1180343	rs1180341	rs755249	rs710913
	Adipose - Subcutaneous		$6.4x10^{-6}$ (-0.33)	$8.7x10^{-6}$ (-0.33)	$5.1x10^{-7}$ (-0.35)	$2.3x10^{-8}$ (0.46)	$3.6x10^{-6}$ (-0.31)

	Adipose - Visceral (Omentum)				$3x10^{-6}$ (-0.44)	$1.5x10^{-9}$ (0.69)	
	Artery - Aorta		$1.1x10^{-6}$ (-0.51)	$3.0x10^{-6}$ (-0.49)	$5.0x10^{-6}$ (-0.47)	$7.7x10^{-10}$ (0.69)	
	Artery - Tibial		$3.8x10^{-9}$ (-0.4)	$7.1x10^{-9}$ (-0.4)	$5x10^{-10}$ (-0.45)	$2.4x10^{-15}$ (0.65)	$1.9x10^{-10}$ (-0.46)
	Breast - Mammary Tissue					$1.4e^{-7}$ (0.55)	
	Cells - Transformed fibroblasts		$4.3x10^{-6}$ (-0.34)	$1.4x10^{-5}$ (-0.33)		$2.6x10^{-11}$ (0.59)	$1.8x10^{-8}$ (-0.41)
	Colon - Sigmoid			$6.5x10^{-6}$ (-0.52)	$8.9x10^{-7}$ (-0.53)		
	Colon - Transverse			$6.1x10^{-6}$ (-0.46)	$3.6x10^{-6}$ (-0.48)		
	Esophagus - Gastroesophageal Junction				$9.7x10^{-7}$ (-0.57)	$3x10^{-6}$ (0.71)	
	Esophagus - Muscularis		$1.4x10^{-9}$ (-0.5)	$8.7x10^{-10}$ (-0.51)	$2.7x10^{-10}$ (-0.53)	$6.5x10^{-9}$ (0.58)	$2.5x10^{-7}$ (-0.43)
	Heart - Atrial Appendage		$1.1x10^{-10}$ (-0.52)	$4.5x10^{-10}$ (-0.53)	$1.3x10^{-9}$ (-0.51)		$3.1x10^{-6}$ (-0.43)
	Lung		$6.6x10^{-6}$ (-0.36)	$8.9x10^{-6}$ (-0.36)	$1.6x10^{-6}$ (-0.37)	$1.7x10^{-9}$ (0.54)	$8.1x10^{-6}$ (-0.33)
	Nerve - Tibial		$1.3x10^{-7}$ (-0.41)	$1.5x10^{-9}$ (-0.47)	$1.2x10^{-7}$ (-0.4)	$1.8x10^{-7}$ (0.44)	
	Spleen		$2.4x10^{-6}$ (-0.68)	$1.1x10^{-6}$ (-0.7)	$2.8x10^{-8}$ (-0.75)		
	Thyroid		$1.7x10^{-7}$ (-0.27)	$3x10^{-8}$ (-0.28)	$1.3x10^{-9}$ (-0.31)	$1.8x10^{-6}$ (0.3)	
